# Anthocyanin synthesis potential in betalain-producing Caryophyllales plants

**DOI:** 10.1007/s10265-021-01341-0

**Published:** 2021-09-03

**Authors:** Masaaki Sakuta, Asuka Tanaka, Kaori Iwase, Mizuki Miyasaka, Sachiko Ichiki, Miho Hatai, Yoriko T. Inoue, Ayumi Yamagami, Takeshi Nakano, Kazuko Yoshida, Setsuko Shimada

**Affiliations:** 1 Department of Biological Sciences, Ochanomizu University, 112-8610 Tokyo, Japan; 2 Graduate School of Biostudies, Kyoto University, 606-8502 Kyoto, Japan; 3 Present Address: Organization for the Strategic Coordination of Research and Intellectual Properties, Meiji University, 214-8571 Kanagawa, Japan; 4 Present Address: Synthetic Genomics Research group, RIKEN Center for Sustainable Resource Science, 230-0045 Yokohama, Kanagawa Japan

**Keywords:** Anthocyanin, Betalain, Caryophyllales, Flavonoid biosynthesis, Production of anthocyanin pigment1, Transcription factor

## Abstract

Although anthocyanins are widely distributed in higher plants, betalains have replaced anthocyanins in most species of the order Caryophyllales. The accumulation of flavonols in Caryophyllales plants implies that the late step of anthocyanin biosynthesis from dihydroflavonols to anthocyanins may be blocked in Caryophyllales. The isolation and characterization of functional dihydroflavonol 4-reductase (DFR) and anthocyanidin synthase (ANS) from Caryophyllales plants has indicated a lack of anthocyanins due to suppression of *DFR* and *ANS*. In this study, we demonstrated that overexpression of *DFR* and *ANS* from *Spinacia oleracea* (*SoDFR* and *SoANS*, respectively) with *PhAN9*, which encodes glutathione S-transferase (required for anthocyanin sequestration) from *Petunia* induces ectopic anthocyanin accumulation in yellow tepals of the cactus *Astrophytum myriostigma*. A promoter assay of *SoANS* showed that the Arabidopsis MYB transcription factor PRODUCTION OF ANTHOCYANIN PIGMENT1 (PAP1) activated the *SoANS* promoter in Arabidopsis leaves. The overexpression of Arabidopsis transcription factors with *PhAN9* also induced ectopic anthocyanin accumulation in yellow cactus tepals. PAP homologs from betalain-producing Caryophyllales did not activate the promoter of *ANS*. In-depth characterization of Caryophyllales PAPs and site-directed mutagenesis in the R2R3–MYB domains identified the amino acid residues affecting transactivation of Caryophyllales PAPs. The substitution of amino acid residues recovered the transactivation ability of Caryophyllales PAPs. Therefore, loss of function in MYB transcription factors may suppress expression of genes involved in the late stage of anthocyanin synthesis, resulting in a lack of anthocyanin in betalain-producing Caryophyllales plants.

**Supplementary Information:**

The online version contains supplementary material available at 10.1007/s10265-021-01341-0.

## Introduction

Plant pigments play essential roles in plant reproduction by generating flower and fruit colors, which recruit pollinators and seed dispersers. Flavonoids and betalains, the main classes of pigments responsible for coloration in plants, also play key roles in plant defense against pathogens and environmental stresses such as drought, salinity, oxidative stress, harmful ultraviolet irradiation (Jain and Gould 2015; Ramakrishna and Ravishankar 2011; Winkel-Shirley 2001). Although anthocyanins, a class of flavonoids, are widely expressed as flower and fruit pigments in higher plants, betalains, comprising yellow betaxanthins and red purple betacyanins, have largely replaced anthocyanins in plants of the order Caryophyllales. To date, the occurrence of anthocyanins in betalain-producing Caryophyllales has not been reported (Mabry 1980; Stafford 1994), and more broadly, anthocyanins and betalains have never been demonstrated to co-exist in a single plant. Although this curious mutual exclusion has been discussed from genetic and evolutionary perspectives (Brockington et al. 2011; Stafford 1994; Timoneda et al. 2019), little is known of its molecular or evolutionary mechanisms. Numerous recent developments have increased our understanding of betalain biosynthesis, including the elucidation of the biosynthetic steps of the betalain pathway and the identification of key genes encoding enzymes of this pathway and transcriptional regulators of betalain synthesis Hatlestad et al. 2012; Lloyd et al. 2017; Sheehan 2020).

Although the molecular mechanism for the absence of anthocyanin biosynthesis in betalain-producing Caryophyllales remains poorly understood, recent studies have elucidated the distinctive evolutionary patterns that typify betalain synthesis. The MBW complex, which contains the MYB and bHLH transcription factors for anthocyanin synthesis, may also participate in the regulation of betalain biosynthesis (Hatlestad et al. 2015). *Beta vulgaris* MYB1 (BvMYB1), an anthocyanin MYB-like protein, regulates the betalain pathway in beets. Silencing BvMYB1 downregulates betalain-biosynthesis genes, and pigment production is upregulated by BvMYB1 overexpression (Hatlestad et al. 2015). Additionally, the availability of tyrosine, a betalain precursor, may affect betalain accumulation (Hirano et al. 1992, 1996; Sakuta et al. 1991). Many species in Caryophyllales possess a canonical form of arogenate dehydrogenase (ADH)β and an additional paralogue of ADHɑ, which has relaxed sensitivity to the negative feedback inhibition by tyrosine in the pathway of tyrosine synthesis (Lopez-Nieves et al. 2018). The relaxation of the tyrosine-mediated feedback inhibition may direct more carbon flow towards tyrosine, and away from phenylalanine biosynthesis and create a surplus of tyrosine at the expense of phenylalanine-derived products such as anthocyanins (Lopez-Nieves et al. 2018). Caryophyllales-specific deregulated ADHɑ enzymes can synthesize higher concentrations of tyrosine, thereby increasing tyrosine availability and resulting in higher betalain accumulation (Grützner et al. 2021; Timoneda et al. 2018). These findings imply an intimate link between the evolution of Caryophyllales-specific deregulated ADHɑ and Caryophyllales-specific betalain biosynthesis.

**Fig. 1:**
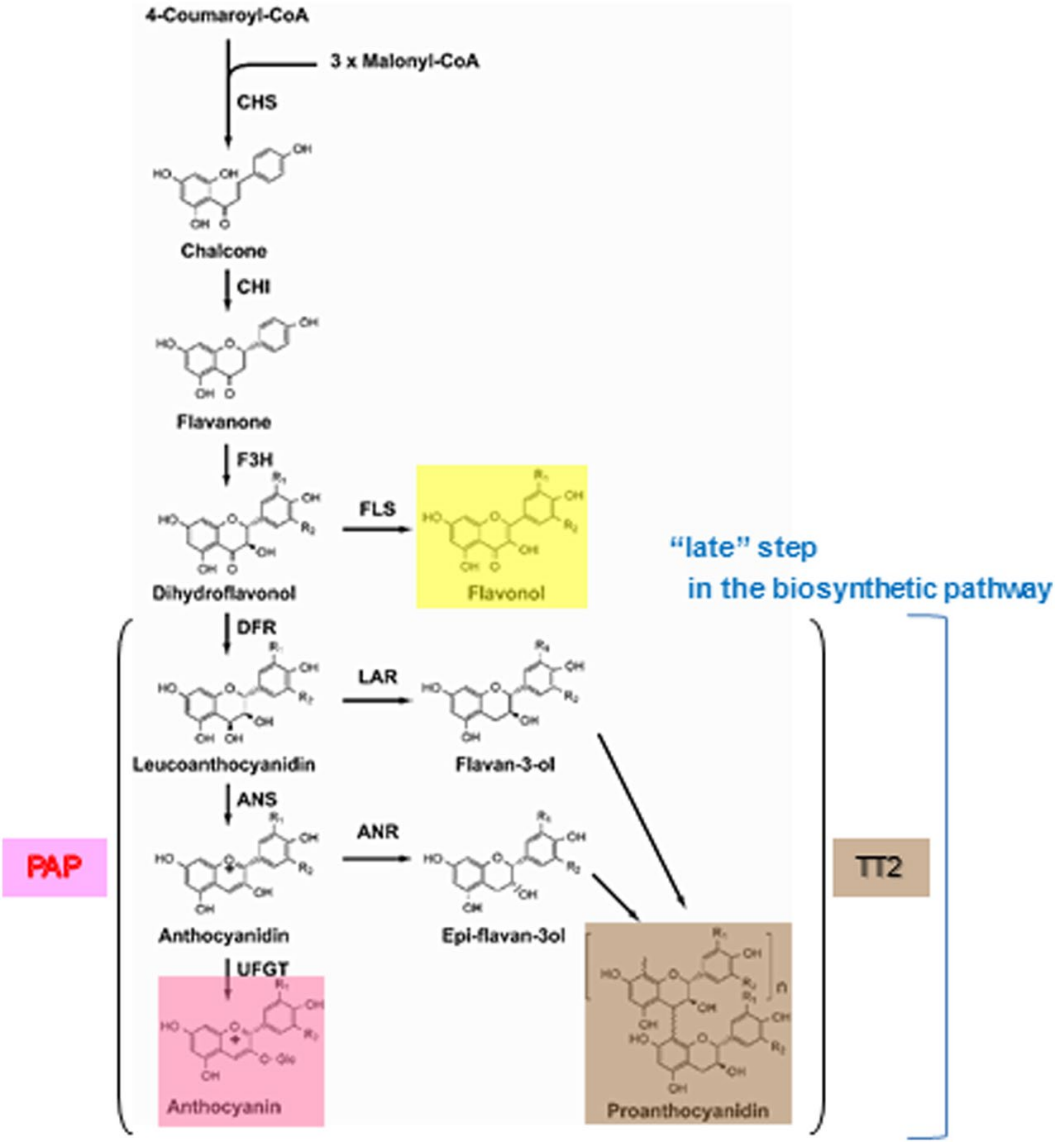
The flavonoid biosynthetic pathway and its regulators. *CHS* chalcone synthase, *CHI* chalcone isomerase, *F3H* flavanone 3-hydroxylase, *FLS* flavonol synthase, *DFR* dihydroflavonol 4-reductase, *ANS* anthocyanidin synthase, *LAR* leucoanthocyanidin reductase, *ANR* anthocyanidin reductase, *UFGT* UDP-glucose: flavonoid 3-O-glucosyltransferase, *PAP* PRODUCTION OF ANTHOCYANIN PIGMENT, *TT2* TRANSPARENT TESTA2

The flavonoid biosynthetic pathway is among the well-studied examples of secondary metabolism in higher plants. Most of genes involved in flavonoid biosynthesis have been cloned and analyzed, and the factors that control gene transcription have been isolated. The regulatory mechanism of flavonoid biosynthesis has been studied in several species (Forkmann and Martens 2001; Holton and Cornish 1995; Koes et al. 2005; Winkel-Shirley 2001). However, in Caryophyllales, anthocyanins have been reported only in the families Caryophyllaceae, Molluginaceae (*sensu stricto*), Kewaceae, Limeaceae, Macarthuriaceae, and Simmondsiaceae (Clement and Mabry 1996; Thulin et al. 2016). Other flavonoids, especially the major flavonols, are common in Caryophyllales (Iwashina 2001). The yellow tepals of *Astrophytum* species contain glycosides (quercetin 3-*O*-galactoside and 3-*O*-rhamnosylglucoside), as well as quercetin, kaempferol, and isorhamnetin in the form of spherical crystals (Iwashina et al. 1988). Dihydroflavonols occur at the branch point in the flavonoid biosynthetic pathway that leads to flavonol and anthocyanin production, implying that anthocyanin biosynthesis from dihydroflavonols may be blocked in betalain-producing Caryophyllales plants (Fig. 1). Dihydroflavonol 4-reductase (DFR) and anthocyanidin synthase (ANS), which are involved in the conversion of dihydroflavonols to anthocyanins have been isolated and their functions identified in two betalain-producing Caryophyllales species, spinach (*Spinacia oleracea*) and pokeweed (*Phytolacca americana*) (Shimada et al. 2004, 2005). Expression profiling revealed that *DFR* and *ANS* were expressed in *S. oleracea* seeds, but not in most other tissues or organs. We presumed that functional copies of *DFR* and *ANS* are maintained for the synthesis of proanthocyanidins, which are accumulated in the seed coat of betalain-producing Caryophyllales, but that transcriptional downregulation in other tissues blocks anthocyanin synthesis in these tissues (Shimada et al. 2004, 2005). Modification of *DFR* and *ANS* cis-regulatory elements may have led to their limited expression, resulting in incomplete anthocyanin synthesis in betalain-producing Caryophyllales (Shimada et al. 2007). Another possibility is loss of function in transcriptional regulators of anthocyanin biosynthetic genes. Transcriptional regulation of flavonoid biosynthesis, including the anthocyanin, flavonol, and proanthocyanidin pathways, is controlled by members of protein families containing R2R3–MYB domains, basic helix–loop–helix (bHLH) domains, and conserved WD40 repeats (WDRs). The combinational interactions of these factors determines the set of genes to be expressed (Baudry et al. 2004; Broun 2005; Koes et al. 2005; Quattrocchio et al. 1998; Ramsay and Glover 2005).

The R2R3–MYB transcription factors TRANSPARENT TESTA2 (TT2) and PRODUCTION OF ANTHOCYANIN PIGMENT1 (PAP1) are positive regulators of genes in the late flavonoid biosynthetic pathway including *DFR* and *ANS* (Yoshida et al. 2010). TT2 contributes to proanthocyanidin synthesis in the seed coat, controlling the expression of *DFR*, *ANS*, and *anthocyanidin reductase* (*ANR*). Proanthocyanidin synthesis involves leucoanthocyanidin reductase (LAR) and ANR. DFR is involved in both routes of proanthocyanidin synthesis (Constabel 2018; Jun et al. 2018) (Fig. 1). *DFR* expression was highly correlated with proanthocyanidin accumulation in cranberry bean seed coat (Freixas Coutin et al. 2017). Because ANS activity is essential for 2,3-*cis*-flavan-3-ol, but not 2,3-*trans*-flavan-3-ol, production, *ANS* mutants of morning glory accumulate proanthocyanidin normally. Thus, ANS is not essential to proanthocyanidin biosynthesis, but is essential to anthocyanin biosynthesis in flowers and stems (Park et al. 2018). PAP1 is specifically responsible for anthocyanin production by regulating the expression of *DFR*, *ANS*, and *UDP-glucose:flavonoid 3-O-glucosyltransferase* (*UFGT*). The characterization and functional analysis of anthocyanin regulators such as *PAP*-like homologs may provide further insight into the lack of anthocyanin synthesis in betalain-producing Caryophyllales. A more detailed analysis of the transcription of late genes in anthocyanin synthesis and characterization of their regulators will further improve our understanding of the mechanisms that regulate flavonoid biosynthesis in betalain-producing Caryophyllales and may reveal why these plants do not produce anthocyanins.

In this study, we investigated the potential for anthocyanin production and examined the regulatory mechanism behind the expression of late genes, especially *ANS* regulated by PAP in betalain-producing Caryophyllales plants, to gain further insight into the diversification of pigments in higher plants. Based on the results of this study, we discuss a possible explanation for the lack of anthocyanin synthesis in betalain-producing Caryophyllales in the context of the evolution of the transcription factors involved in anthocyanin synthesis.

## Materials and methods

### Plant materials

*Arabidopsis thaliana* (Arabidopsis) ecotype Columbia (Col-0) was used as the WT plant. The Arabidopsis T-DNA insertional *tds4* (*ans* mutant) was obtained from the Arabidopsis Biological Resource Center (accession no. SALK_073183). Plants were genotyped through PCR of genomic DNA using a set of *ANS*-specific primers (ANS-GT-L and ANS-GT-R) and a T-DNA left border primer, as previously described (http://signal.salk.edu/tdnaprimers.2.html). Seeds of *Portulaca grandiflora* and *Basella alba* and nursery plants of *Mammillaria zeilmanniana* (cv. Tsukikage-maru) were purchased from Sakata Seeds. *Astrophytum myriostigma* was kindly provided by Mr. Shimada, Gunsenen. Seeds were germinated on 1/2 MS medium containing 0.8 % phytoagar with 1.5 % sucrose, and then transferred to soil. Arabidopsis plants were grown at 22 °C under a 16-h light/8-h dark cycle. *P. grandiflora* and *B. alba* were maintained at 27 °C under the same light cycle. Both cactus species (*A. myriostigma* and *M. zeilmanniana*) were grown in a greenhouse.

### Complementation analysis

To generate the *35 S::SoANS* construct, the *SoANS* ORF was amplified from the pMAL: *SoANS* clone (Shimada et al. 2005) using PCR with the primers spiANS-gw-F and spiANS-gw-R (Table S1). To generate the *35 S*::*PfANS* construct, the ORF of *PfANS* was amplified from a pLMK1 clone (Saito et al. 1999) kindly provided by Dr. Kazuki Saito, Chiba University, using PCR with the primers perANS-gw-F and perANS-gw-R. Each PCR product was cloned via directional TOPO cloning into the entry vector pENTR/D-TOPO (Invitrogen). After sequencing, the fragment was transferred into the pGWB2 binary vector, kindly provided by Dr. Tsuyoshi Nakagawa, Shimane University, via an LR recombination reaction. The resulting plasmid was transformed into *tds4* using the floral dip method. T2 seeds of three independent transgenic lines were germinated in triplicate on 5 % sucrose. Six-day-old seedlings were extracted overnight at 4 °C in 100 µL of 1% (v/v) HCl in methanol. Anthocyanin contents were estimated by measuring the absorbance at 530 nm and normalized for the fresh weight of the tissue used. Total RNA from 13-day-old seedlings of *tds4* and *tds4* transformed with *35 S*::*SoANS* was subjected to RT-PCR. The primers ANS-F1/ANS-R3, spiANS-gw-F/spiA, perANS-F/perANS-gw-R, and Actin-F1/Actin-R1 were used to amplify *AtANS*, *SoANS*, *PfANS*, and *ACTIN*, respectively (Table S1). PCR was performed for 30 cycles (94 °C for 15 s, 57 °C for 30 s, and 72 °C for 1 min).

### Promoter::*GUS* assay

For functional analysis of the *SoANSpro* in Arabidopsis, plasmids for the GUS assay were constructed by inserting a fragment of the *SoANSpro* into the multiple cloning sites of the pBI101 vector (Shimada 2007). The fragments were generated by PCR from *S. oleracea* genomic DNA using the primer set SoANSs2 and SoANSas2, with restriction enzyme sites HindIII and BamHI added, respectively (Table S1). The fragments were ligated in-frame to the pBI101 vector, and the resulting plasmid was transformed into Col-0 using the floral dip method.

### GUS staining

Arabidopsis seedlings expressing the *SoANSpro*::*GUS* fused reporter gene were histochemically stained according to the method of Ito and Fukuda (2002), with minor modifications. Briefly, the seedlings were treated with acetone for 20 min. After rinsing with water, the plants were incubated in a solution containing 0.4 mg/mL X-Gluc, 0.5 mM K_4_[Fe(CN)_6_], 0.5 mM K_3_[Fe(CN)_6_], and 1 mM ethylenediamine tetraacetic acid (EDTA) in 50 mM phosphate buffer overnight at 37 °C. After rinsing with ethanol and water, blue staining was observed in each seedling.

### Transient expression assay

Effector plasmids including *AtTT2, AtTT8* (*TRANSPARENT TESTA8*), *AtTTG1* (*TRANSPARENT TESTA GLABRA1*), *PhAN2* (*ANTHOCYANIN2*) and reporter plasmids including *AtANSpro*, *AtDFRpro*, *SoANSpro*, and *SoDFRpro*, were previously constructed (Quattrocchio et al. 1998; Shimada et al. 2007). For the AtPAP1 expression vector, the ORF of *AtPAP* was amplified using PCR with AtPAP-S-Sal I, AtPAP-AS-Not I, KOD DNA polymerase; the fragment was subcloned into the *35 S*::*sGFP(S65T)* vector, replacing the *GFP* gene. Particle bombardment of Arabidopsis leaf cells using effector and reporter constructs with *35 S*::*RUC* and dual luciferase assay was performed as described by Yoshida et al. (2008). Arabidopsis rosette leaves were incubated for 20 min on 50 % MS plates containing 1.5 % (w/v) sucrose and 0.25 M mannitol. The cells were subjected to bombardment at a pressure of 450 psi under a vacuum of 26 mm Hg. After transformation, the tissues were incubated in the dark for 20 h at 25 °C. For the luciferase assay, leaves were frozen with liquid nitrogen and ground in cell culture lysis reagent buffer containing 100 mM potassium phosphate, pH 7.8, 1 mM EDTA, 7 mM 2-mercaptoethanol, 1 % (v/v) Triton X-100, and 10 % (v/v) glycerol. The extracts were subjected to the luciferase assay. LUC and RUC activity were quantified using a PicaGene Dual Luminescence Kit (Toyo Ink, Tokyo, Japan). Luciferase activity was detected using an AB-2250 luminescencer (ATTO, Tokyo, Japan) for 30 s at room temperature. Relative reporter gene activity was calculated as LUC/RUC; the arithmetical average and standard deviation of three replicates are reported.

The plasmids for transient expression assay of *Astrophytum myriostigma* tepals were constructed by fusing the ORFs of *SoDFR* (Shimada et al. 2004), *SoANS* (Shimada et al. 2005), and *PhAN9* (Mueller et al. 2000) to the *CaMV 35 S* promoter by replacing *sGFP* in the *35 S::sGFP(S65T)* vector. In the transient assay of *Astrophytum myriostigma*, tepals were detached from buds (longest diameter, 2 cm) and placed on MS medium containing 0.24 M mannitol and 9 % agar at pH 6.0. The tepals on MS medium plates were subjected to bombardment at a pressure of 450 psi under a vacuum of 26 mm Hg. After transformation, the tissues were incubated under fluorescent light at 25 °C. Tepals with red cells were placed under a microscope equipped with a micro-spectrophotometer (JASCO MSV-370T; JASCO, Tokyo, Japan) and their absorption spectra in the visible region (400–800 nm) were measured. For alkali treatment, 25 % ammonia water was dropped on the slide glass and color changes were observed.

### Isolation of *PAP* homologs from Caryophyllales plants

The methods for genomic DNA isolation, total RNA isolation, cDNA synthesis have been previously described (Shimada 2007). To detect conserved *PAP* homologs, we designed two sets of primers for nested PCR (PAPlikesearch1F and PAPlikesearch1R for the first PCR; PAPlikesearch2F and PAPlikesearch2R for the second PCR) from the most conserved regions of known *PAP* sequences (Niu et al. 2010; Pattanaik et al. 2010). The genome sequence of *PgPAP* was determined via TAIL PCR using the TAIL primers listed in Table S1. We used an LA PCR *in vitro* Cloning Kit (TaKaRa, Kyoto, Japan) for genome sequence analysis of *PgPAP*, according to the manufacturer’s instructions. The cDNAs of *BaPAP* and *MzPAP* were cloned by rapid amplification of cDNA ends (RACE) as previously described (Shimada et al. 2005). For 3’-RACE of *BaPAP* cDNA, the primers ODTA and BaPAPlike3’ RACE 1F were used for the first PCR, and BaPAPlike3’ RACE 2F for the second PCR. For 5’-RACE of *BaPAP* cDNA, the primers AUAP (dG) and BaPAPlike5’ RACE 1F were used for the first PCR, and BaPAPlike5’ RACE 2F for the second PCR. For 3’-RACE of *MzPAP* cDNA, the primers ODTA and MzPAPlike3’ RACE 1F were used for the first PCR, and MzPAPlike3’ RACE 2F for the second PCR. For 5’-RACE of *MzPAP* cDNA, the primers AUAP(dG) and MzPAPlike5’ RACE 1F were used for the first PCR, and MzPAPlike5’ RACE 2 F for the second PCR (Table S1). Full-length cDNAs were cloned via PCR using the primer sets BaPAP-F-SalI and BaPAP-R-Not for BaPAP, and MzPAP-F-SalI and MzPAP-R-NotI for MzPAP (Table S1).

### Semi-quantitative RT-PCR

Total RNA from each part of *Portulaca grandiflora* was used for first-strand cDNA synthesis. The quantity of each template was adjusted for roughly equal amplification of actin cDNA. RT-PCR was performed using 0.5 pmol of each gene-specific primer with Taq DNA polymerase for a final volume of 20 µL. The primers PgPAP-ACTIN-F/PgPAP-ACTIN-R, RT-PCR_PgPAP-F/RT-PCR_PgPAP-R were used to amplify ACTIN and PgPAP, respectively (Table S1).

### Site-directed mutagenesis

Mutated *PgPAPs* were constructed using a KOD-Plus Mutagenesis Kit (TOYOBO, Osaka, Japan) according to the manufacturer’s instructions, using the mutated primers V4GF, F47LF, H66LF, K69RF, Y74LF, and S83GF as forward primers and V4GR, F47LR, H66LR, K69RR, Y74LR, and S83GR as reverse primers (Table S1).

### DNA sequencing

Nucleotide sequences were determined using a BigDye Terminator v. 3.1 Cycle Sequencing Kit and an ABI Prism 3100-Avant genetic analyzer (Applied Biosystems, Foster City, CA, USA).

## Results

### Complementation of Caryophyllales *ANS* in the Arabidopsis *ans* mutant shows ectopic accumulation of anthocyanins

**Fig. 2:**
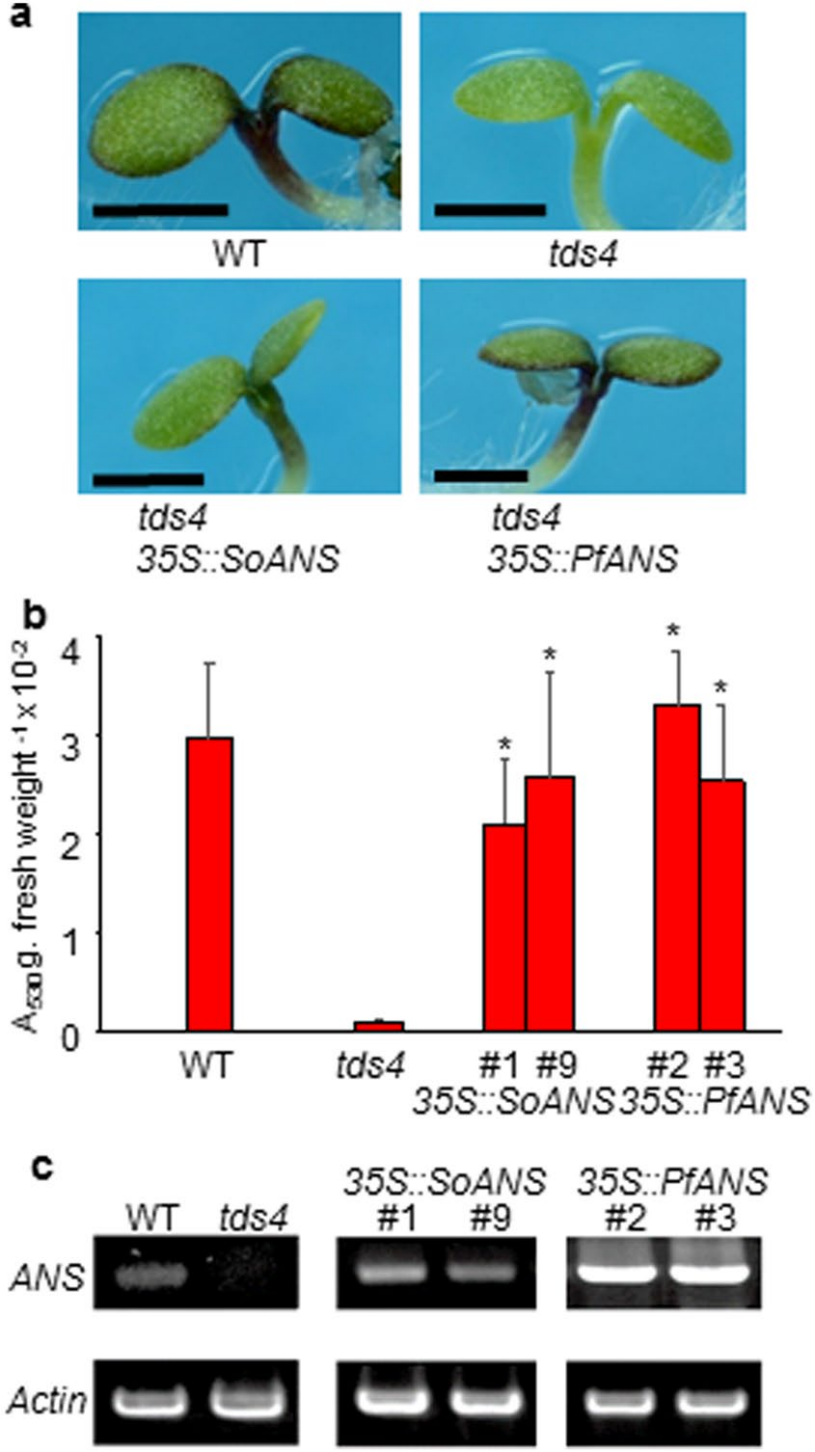
Analysis of *ANS* overexpression in an Arabidopsis *ans* mutant. **a** Anthocyanin phenotypes of seedlings of wild-type (WT) Arabidopsis, Arabidopsis *ans* mutant *tds4*, and Arabidopsis *ans* mutant *tds4* lines transformed with *35 S::SoANS*, *35 S::Pf ANS.* Bars represent 1.0 mm. **b** Anthocyanin levels in seedlings of *ANS*-overexpressing lines, Arabidopsis *ans* mutant *tds4*, and WT *Arabidopsis* grown on 1/2 Murashige and Skoog (MS) medium containing 5 % sucrose (mean + s.d., *P < 0.05 by Student’s *t* test, compared with *tds4*, *n* = 3). **c** Transcript levels of *ANS* in seedlings of the Arabidopsis WT, *ans* mutant *tds4*, and Arabidopsis *ans* mutant *tds4* lines transformed with *35 S*::*SoANS* and *35 S*::*PfANS*

Isolation and *in vitro* functional identification of *ANS* genes from *S. oleracea* and *P. americana* have been previously reported (Shimada et al. 2005). We further conducted *in vivo* molecular complementation of the Arabidopsis *ans* mutant line (*tds4*) with *S. oleracea* *ANS* (*SoANS*). The *ANS* gene of *Perilla frutescens* (*PfANS*; Saito et al. 1999) was used as a positive control in the complementation assays. Seeds of Arabidopsis Columbia, *tds4*, and transgenic plants were plated on half-strength Murashige and Skoog (MS) agar medium containing 5 % sucrose to induce anthocyanin biosynthesis (Solfanelli et al. 2006) (Fig. 2). Anthocyanin accumulation in seedlings was measured at 6 days after germination. Compared with the *tds4* mutant, increased anthocyanin accumulation was observed in wild-type (Columbia) (WT) and transgenic plants (Fig. 2a, b). *ANS* expression was detected in the WT and overexpression lines of *SoANS* and *PfANS*, but not in *tds4*, using semi-quantitative reverse-transcription polymerase chain reaction (RT-PCR) (Fig. 2c). These results indicated that ectopic expression of *SoANS* in *tds4* induced significant anthocyanin accumulation, although lower than that of *PfANS* (Fig. 2b, c).

### Overexpression of *DFR* and *ANS* is sufficient to induce anthocyanin accumulation in *Astrophytum myriostigma* yellow tepals in the presence of the anthocyanin carrier protein

**Fig. 3:**
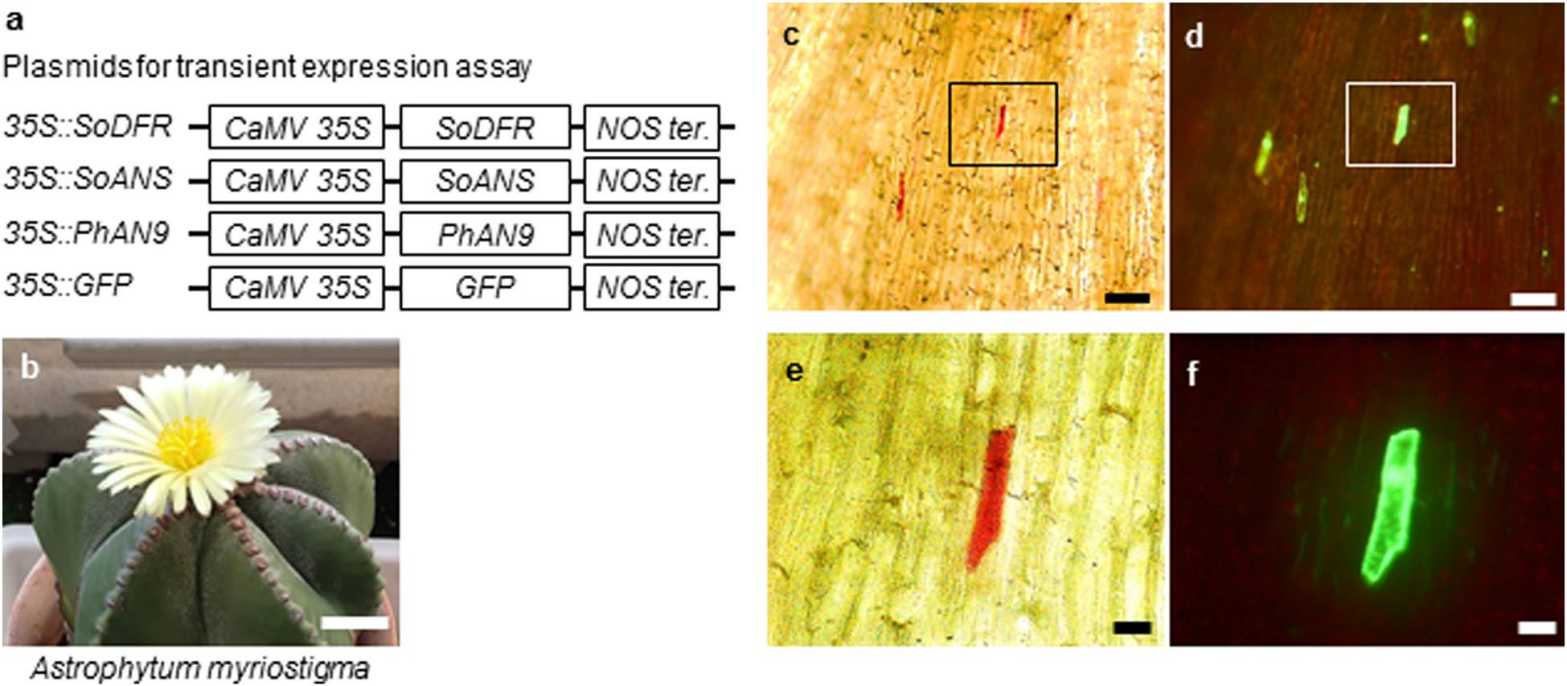
Ectopic anthocyanin accumulation in *Astrophytum myriostigma* yellow tepals by overexpression of *SoDFR* and *SoANS* with *PhAN9*. **a** The plasmid constructs for transient expression assays are shown. **b** A flower of *Astrophytum myriostigma*; the bar represents 10 mm. Microscopic observation of (**c**) red cells and (**e**) a single cell accumulating anthocyanins, expressing green fluorescent protein (GFP; **d**, **f**) after bombardment with *35 S::SoDFR*, *35 S::SoANS*, *35 S::PhAN9*, and *35 S::GFP*. Bars in **c** and **d** represent 50 μm. Bars in **e** and **f** represent 10 μm

In *Astrophytum* species, yellow tepals contain several flavonols, including glycosides (quercetin 3-*O*-galactoside and 3-*O*-rhamnosylglucoside), quercetin, kaempferol, and isorhamnetin, in the form of spherical crystals (Iwashina et al. 1988). Dihydroflavonols occur at the branch point in the flavonoid biosynthetic pathway leading to flavonols and anthocyanins (Fig. 1). Therefore, we conducted a biochemical complementation study to determine whether metabolic flow shifts to anthocyanin synthesis through overexpression of *DFR* and *ANS* in *Astrophytum myriostigma* yellow tepals (Fig. 3a, b). After 3 days of incubation, transient overexpression of the genes *SoDFR*, *SoANS*, and *PhAN9*, which encodes glutathione S-transferase (GST), required for anthocyanin sequestration (Mueller et al. 2000), through shotgun transformation of *Astrophytum myriostigma* yellow tepals produced red spots with a maximum wavelength of 530 nm (Fig. 3c, e, Fig. S1a). Red pigment was accumulated in vacuoles (Fig. 3c, e) and green fluorescent protein (GFP) fluorescence was observed in the nuclei and cytosol around vacuoles (Fig. 3d, f). Red cells were not observed following particle bombardment of *SoDFR* and *SoANS* without *PhAN9*. As red pigments, both anthocyanin and betacyanin exhibit similar absorption spectra in the visible region (400–800 nm). Vast quantities of molecular species of anthocyanins are created through the combination of aglycones with sugars and organic acid moieties; each molecular species has an individual retention time that can be identified through high-performance liquid chromatography analysis. Therefore, it is difficult to analyze pigments within a single cell using instrumental analysis. Color changes induced by alkali treatment are a simple and effective method for distinguishing anthocyanins from betacyanins (Venkataraman 1962). Under alkaline conditions, anthocyanins turn blue and betacyanins turn brownish yellow (Fig. S1d). The change of a cell from red to blue following alkali treatment indicates that the color of the transformed cell is produced by anthocyanins (Fig. S1b, c).

### The *ANS* promoter of betalain-producing Caryophyllales is activated by Arabidopsis transcription factors

In a previous study, we isolated and analyzed *DFR* and *ANS* promoters of the Caryophyllales (*S. oleracea* and *P. americana*), and implied that suppression or limited expression of *ANS* may be responsible for the lack of anthocyanin synthesis in betalain-producing Caryophyllales (Shimada et al. 2005). To gain further insight into the control of *ANS* expression in betalain-producing Caryophyllales plants, we examined the function of the *ANS* promoter of betalain-producing Caryophyllales in Arabidopsis. The *SoANS* promoter region (*SoANSpro*) was fused to *β-glucuronidase* (*GUS*) and introduced into Arabidopsis. *GUS* expression was observed in anthocyanin-accumulating tissues in seedlings (Fig. S2). The results imply that *SoANSpro* can be activated by transcription factors in Arabidopsis.

**Fig. 4:**
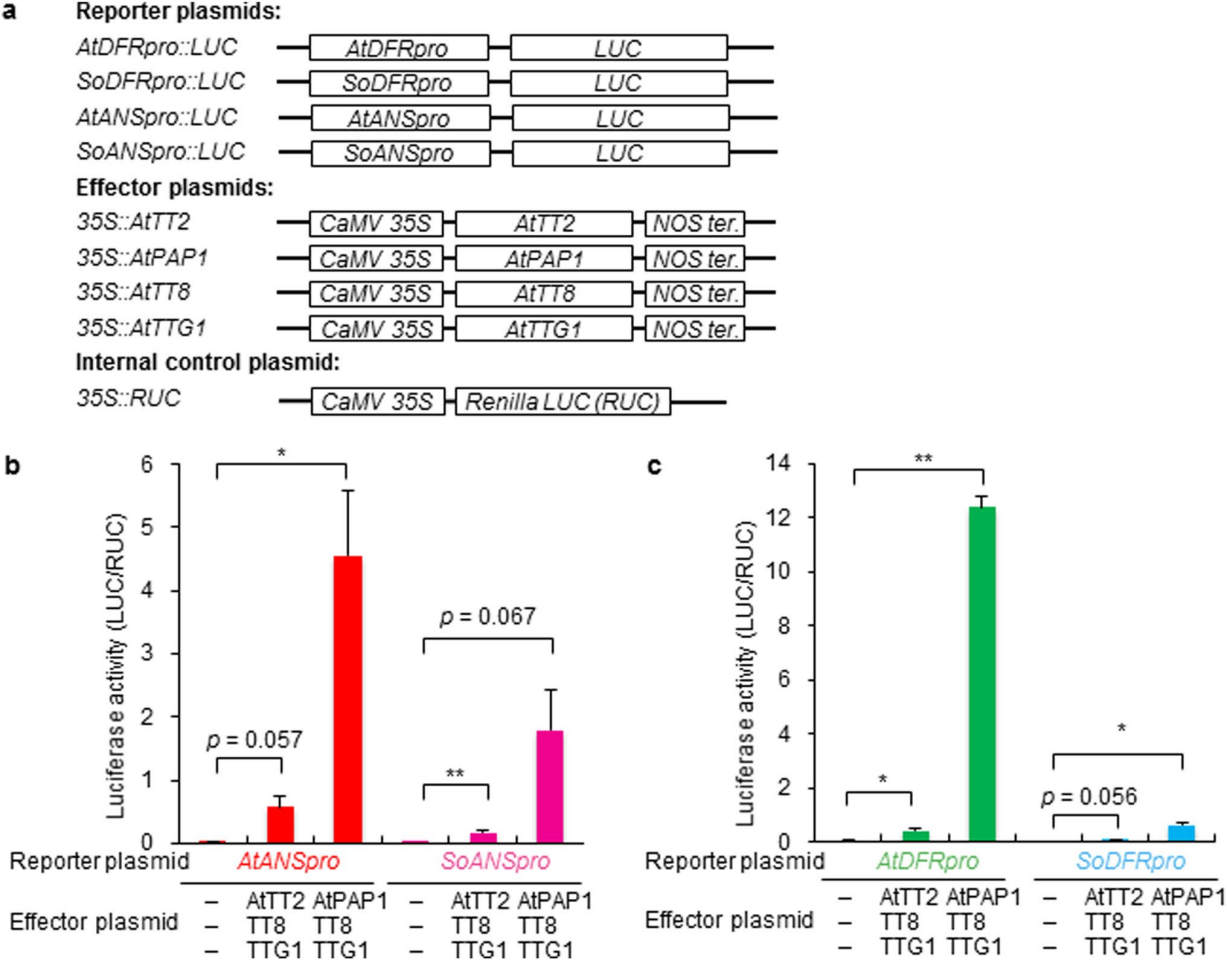
*SoANS* and *SoDFR* promoter assays in Arabidopsis. **a** The reporter and effector plasmid constructs used are shown. Transactivation of Arabidopsis PAP1 or TT2 with TT8 and TTG1 on reporter constructs of **b** *AtANS promoter* and *SoANS promoter* and **c** *AtDFR promoter* and *SoDFR promoter*, which contain the *firefly luciferase* (*LUC*) gene as a reporter gene, were analyzed in particle-bombarded Arabidopsis leaf cells. The reporter gene activity, measured as LUC enzyme activity, is expressed in arbitrary units and normalized to *Renilla* luciferase (RUC) activity as expressed by the co-bombarded internal control plasmid *CaMV35S::RUC* (mean ± s.d., ^**^P < 0.01, ^*^P < 0.05 by Student’s *t *test, *n* = 3)

In several plants, regulatory complexes have been shown to involve WDRs, bHLH, and MYB in anthocyanin and proanthocyanidin biosynthetic processes (reviewed in Broun 2005; Koes et al. 2005; Ramsay and Glover 2005). To assess the functionality of the *SoANSpro*, the transactivation abilities of these factors were examined in a dual luciferase experiment using a *SoANSpro*::*LUC* (*firefly luciferase*) construct as the reporter (Fig. 4a). For comparative analysis of the *ANS* promoter of betalain-producing Caryophyllales and that of anthocyanin-producing species, the *AtANS* promoter (*AtANSpro*) was also used as a control in this assay. Effector constructs containing prospective transcriptional regulator genes for *AtANS*, and for internal controls containing the *Renilla luciferase* (*RUC*) gene driven by the *cauliflower mosaic virus (CaMV)* *35 S* promoter, were co-bombarded with reporter constructs into Arabidopsis leaves. Since the PAP1/ TT8 and ENHANCER OF GLABRA3 (EGL3)/ TTG1 complex appear to be an innate combination for anthocyanin regulation in Arabidopsis (Baudry et al. 2006), combinations of AtPAP1, AtTT8, and AtTTG1 were first examined to test for activation of the *SoANSpro*. AtPAP1 significantly activated the *SoANSpro* with AtTT8 and AtTTG1. The transactivation ability of AtPAP/AtTT8/AtTTG1 for the *SoANSpro* was less than half of that for *AtANSpro*, but more than threefold higher than that of co-expression of AtTT2, which activates *AtANS* as a proanthocyanidin regulator, with AtTT8 and AtTTG1 for the *AtANSpro* (Fig. 4b). The *SoDFR* prompter (*SoDFRpro*) is also activated by AtPAP/AtTT8/AtTTG1. The transactivation of the *SoDFRpro* by AtPAP/AtTT8/AtTTG1 was less than one tenth that of *AtDFR*, but more than twofold higher than that by co-expression of AtTT2, which activates *AtDFR* as a proanthocyanidin regulator, with AtTT8 and AtTTG1 (Fig. 4c). No sole reporter construct resulted in significant LUC activity (Fig. S3).

### Ectopic expression of anthocyanin regulators induces anthocyanin accumulation in *Astrophytum myriostigma* yellow tepals in the presence of an anthocyanin carrier protein

**Fig. 5:**
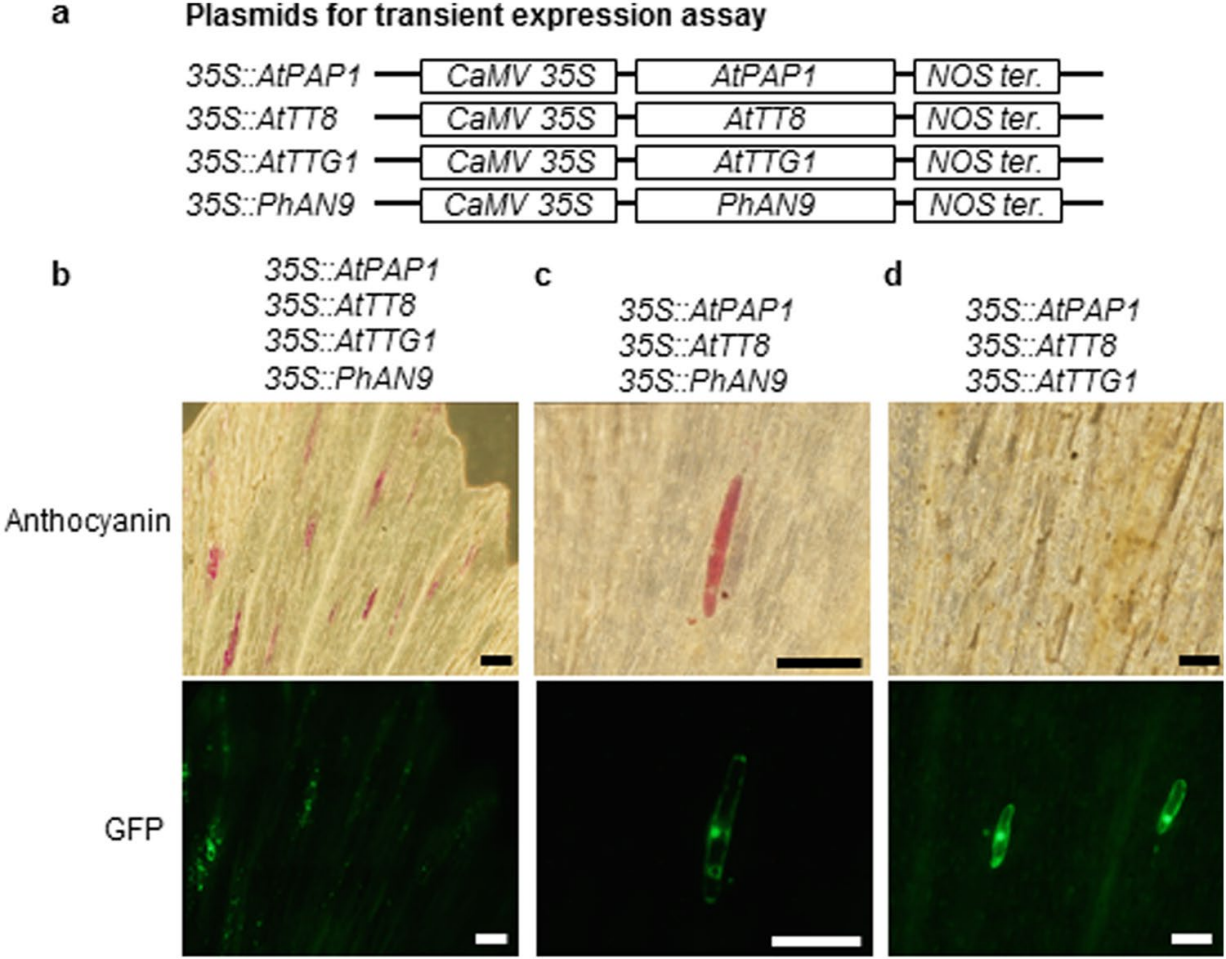
Ectopic anthocyanin accumulation in *Astrophytum myriostigma* yellow tepals induced by overexpression of Arabidopsis transcription factors. **a** The plasmid constructs for transient expression assays. Anthocyanin phenotype (upper panels) and GFP expression (lower panels) in an overexpressor of Arabidopsis transcription factors with (**b**, **c**) or without (**d**) *PhAN9*. Bars represent 100 μm

Based on promoter analysis of *SoANS* and *SoDFR*, which are activated by transcription factors in Arabidopsis, transient overexpression of heterologous transcription factors that regulate anthocyanin synthesis was attempted in *Astrophytum myriostigma* yellow tepals (Fig. 5a). After 3 days of incubation, transient overexpression of *PAP1*, *TT8*, *TTG1*, and *PhAN9* produced red spots in tepals (Fig. 5b). The overexpression of *PAP1*, *TT8*, and *PhAN9* without *TTG1* (Fig. 5c) or that of *PAP1* and *EGL3* instead of *TT8* (Baudry et al. 2006; Gonzalez et al. 2007) and *PhAN9* also induced anthocyanin accumulation, whereas the elimination of *PhAN9* from transcription factors did not lead to anthocyanin accumulation (Fig. 5d).

### Betalain-producing Caryophyllales plants possess *PAP* homologs

**Fig. 6:**
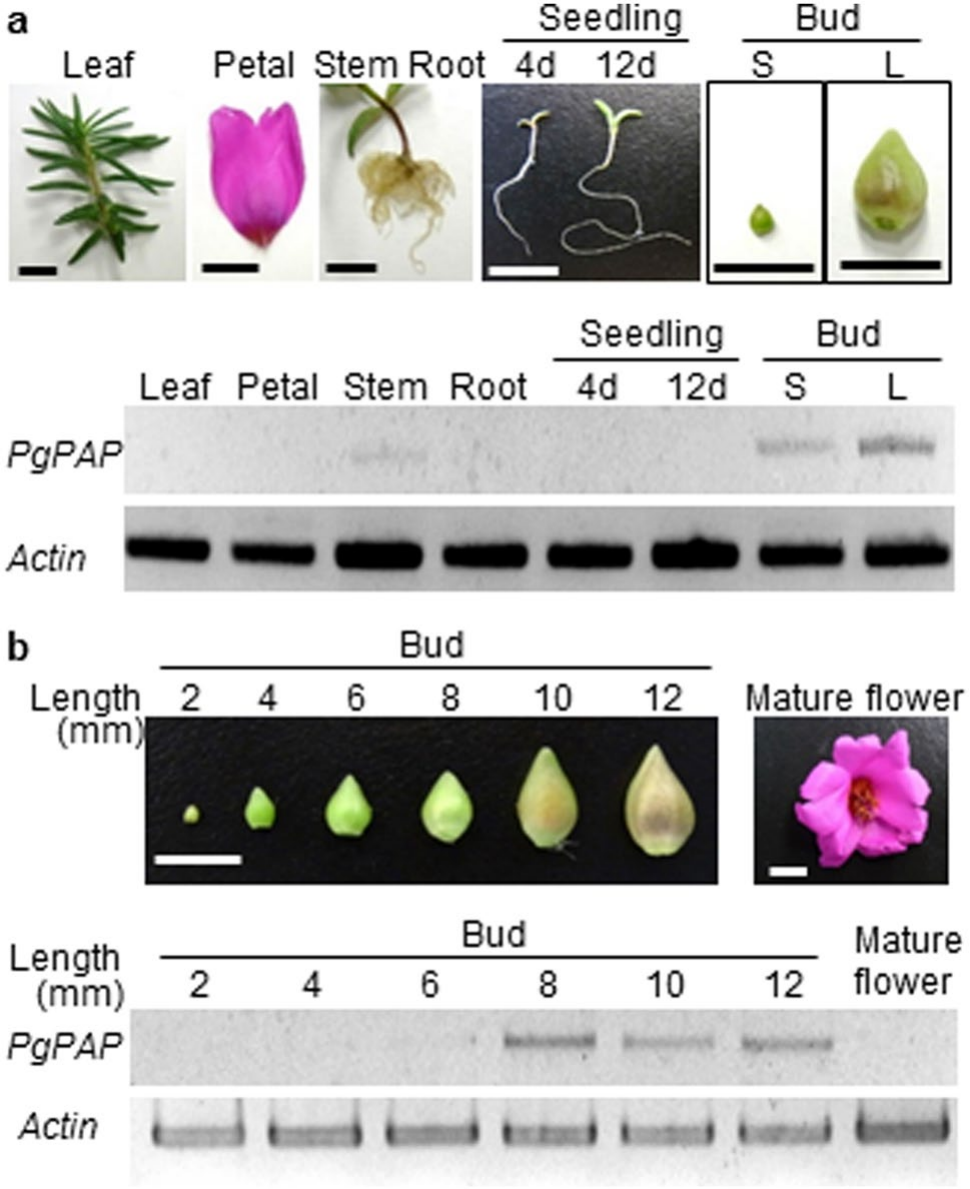
Expression profiles of *PgPAP*. **a** Expression of *PgPAP* in various organs of *Portulaca grandiflora*. Total RNA extracted from seedlings at 4 and 12 days after germination, and from leaves, petals, stems, roots, and buds was used for semi-quantitative reverse-transcription polymerase chain reaction (RT-PCR). **b** RT-PCR analysis of *PgPAP* transcript levels during flower bud development. Bars represent 10 mm

**Fig. 7:**
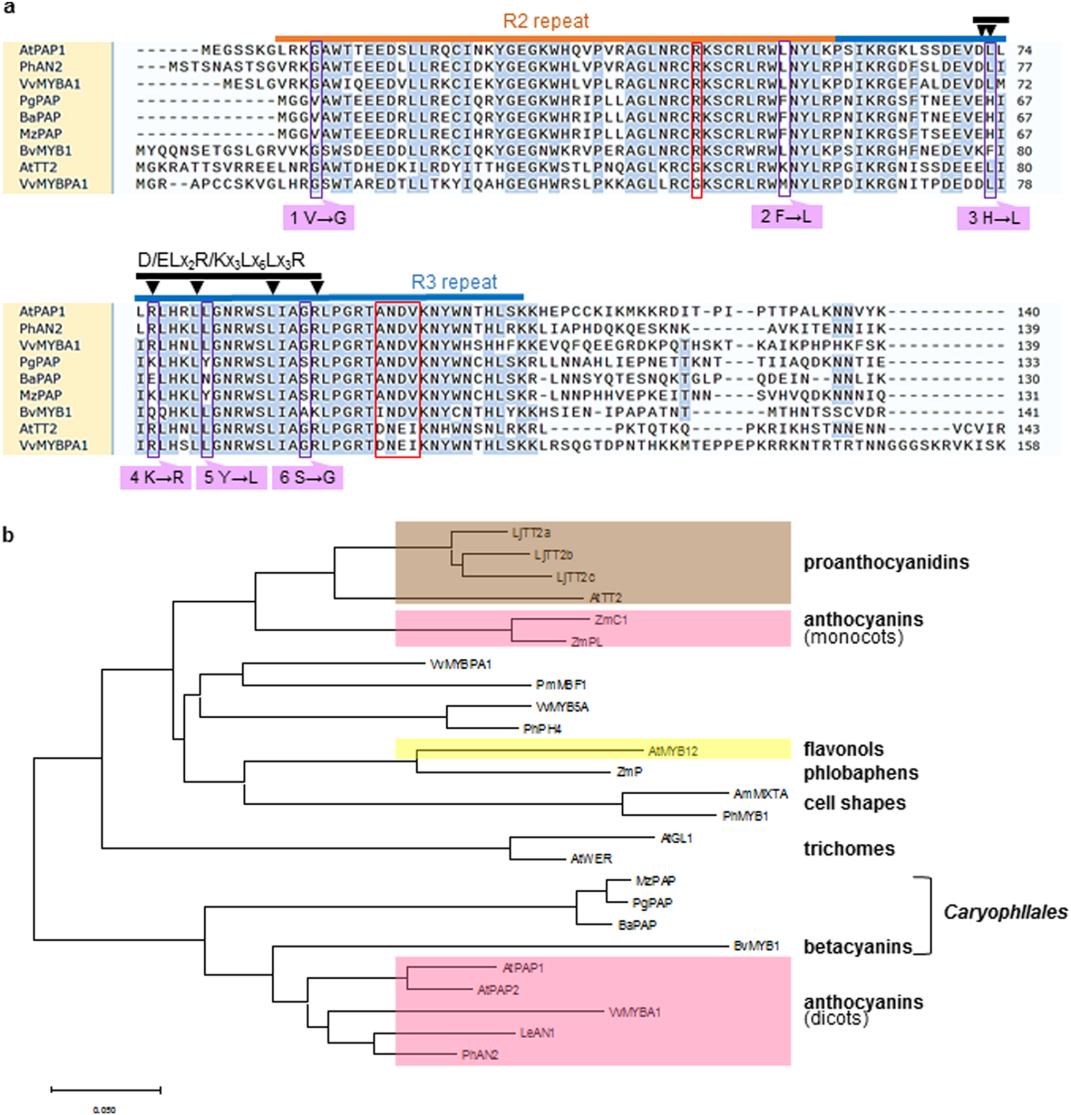
Characterization of PAP homologs from Caryophyllales. **a** Alignment of deduced amino acid sequences of MYB-type transcriptional regulators and illustration of site-directed mutagenesis of PAPs of betalain-producing plants. Bars indicate the R2 and R3 repeats of the MYB domain. Red boxes indicate conserved motifs found in PAP homologs. Arrows indicate amino acid residues that determine interactions with bHLH-related factors (Grotewold et al. 2000; Zimmermann et al. 2004). Black boxes indicate amino acid residues common among PAPs of anthocyanin-producing plants that were replaced in PAPs of betalain-producing plants. Purple boxes show amino acid residues replaced by site-directed mutagenesis. **b** Phylogenetic analysis of the relationships among R2R3 domains from MYB-related proteins. To construct the tree, we used only the R2R3–MYB domain sequence of each selected MYB-related protein. Plant MYB transcription factors were selected from the GenBank database. Accession numbers are listed in Table S2. The functions of some proteins are indicated in bold. The bar represents 0.05 substitutions per site

We attempted to detect *PAP* homologs in the *Portulaca grandiflora* genome using nested PCR with degenerate primers designed based on the most highly conserved regions found through the alignment of *PAP* nucleotide sequences from different plants. Subsequent thermal asymmetric interlaced (TAIL) PCR and long-and-accurate (LA) PCR identified the genome sequence of *P. grandiflora* containing the *PAP* conserved region. Fig. S4a shows the genomic organization of the identified sequences, which consisted of three exons and two introns (GenBank accession no. MW192237). The isolated open reading frame (ORF), named *PgPAP* (GenBank accession no. MW192234), showed significant similarity to *AtPAP1* in the R2R3–MYB domain (Fig. S5). Sequences of the isolated *PgPAP* promoter are shown in Fig. S4b. Analysis of the promoter sequences using the Plant Cis-acting Regulatory DNA Elements (PLACE) database (https://www.dna.affrc.go.jp/PLACE/?action=newplace) revealed several elements to which transcriptional factors can bind in the *PgPAP* promoter. In the promoter, we identified four POLLEN1LELAT52 motifs involved in pollen-specific transcription, two MRE motifs involved in light regulation, and two RY motifs that are conserved in seed-specific promoters, as well as Skn-1, which is involved in endosperm formation, a gibberellin responsive element (GARE), and a methyl jasmonate responsive motif (TCAGC). To gain an overview of the expression profiles of *PgPAP* in *P. grandiflora*, we performed RT-PCR using total RNA prepared from various tissues and/or organs including seedlings at different stages of development (Fig. 6). The *PgPAP* transcripts were not detected in any of these samples, except in buds (Fig. 6a). The levels of *PgPAP* transcripts increased with increasing bud size and decreased with flowering (Fig. 6b). We also isolated and characterized the *PAP* homolog cDNAs *BaPAP* (GenBank accession no. MW192236) and *MzPAP* (GenBank accession no. MW192235) from the betalain-producing Caryophyllales species *Basella alba* and *Mammillaria zeilmanniana*, respectively. The alignment of predicted amino acid sequences in PAP homologs of betalain-producing Caryophyllales and anthocyanin-producing plants was well conserved except at both ends of the sequences (Fig. S5). The PAP homologs of the betalain-producing Caryophyllales species contained the R2R3 repeat DNA-binding conserved domains located in tandem at the N-terminal regions, which contain the motif [D/E]Lx2[R/K]x3Lx6Lx3R for interaction with the bHLH protein (Grotewold et al. 2000; Zimmermann et al. 2004), with the exception of [D/E]Lx2, which changed to [D/E]Hx2 in PAP homologs of Caryophyllales isolated in this study (Fig. 7a). The deduced amino acid sequence of the R2R3–MYB domain of the PgPAP homolog shared 73 % identity with AtPAP, 78 % with PhPAP, 77 % with NtAN2, and 72 % with VvMYBA1. Notably, amino acid residues R and ANDV, which are common to PAPs (Heppel et al. 2013; Lin-Wang et al. 2010) were conserved in betalain-producing Caryophyllales PgPAP homologs isolated in this study (Fig. 7a). A phylogenetic tree created from the R2R3–MYB domains using the neighbor-joining method (Saitou and Nei 1987) showed that the PAP homologs of betalain-producing Caryophyllales plants were clustered with PAPs from anthocyanin-synthesizing dicot plants (Fig. 7b).

### Substitution of amino acid residues recovers the transactivation ability of betalain-producing Caryophyllales PAP homologs to the same level as that of PhAN2

**Fig. 8:**
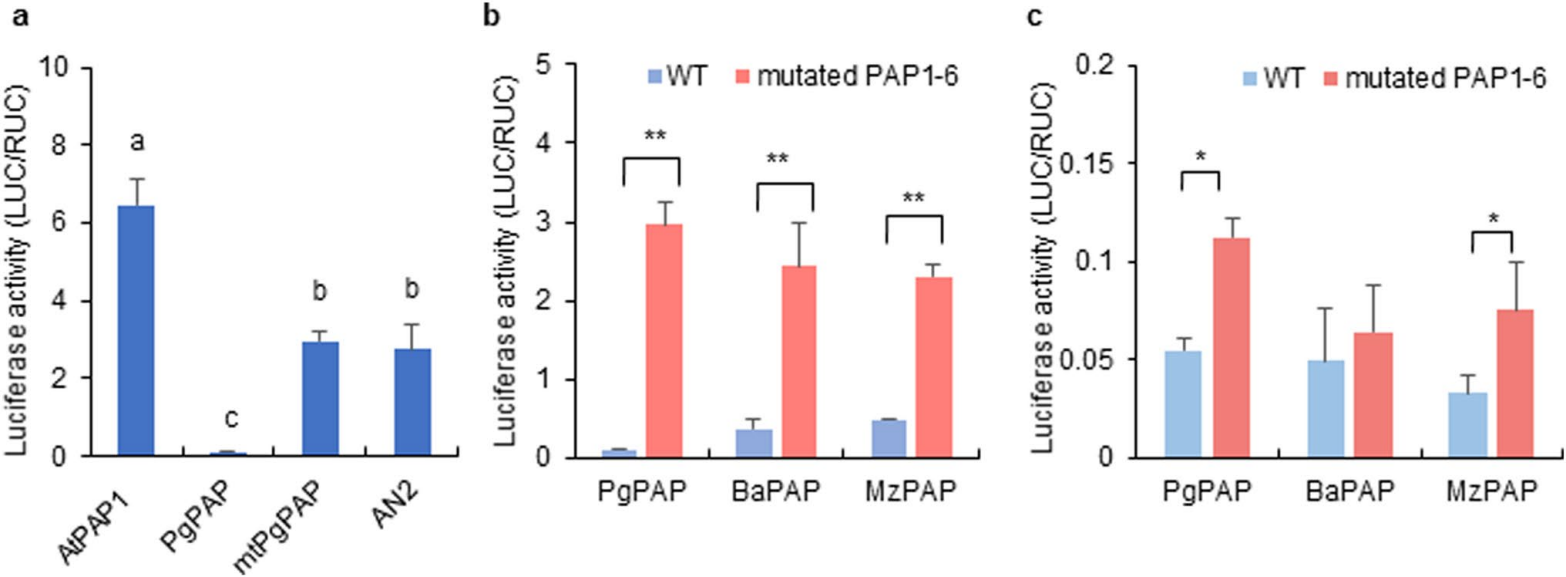
Activation of *AtANS* and *SoANS promoter* by ectopic expression of PAPs. **a** Activation of *AtANS promoter* by AtPAP, PgPAP, PhAN2, and mutated PgPAP1–6 (mtPgPAP) (mean ± s.d., *P* < 0.05 by Tukey’s test, *n* = 3). **b** Activation of *AtANS promoter* by PAPs of betalain-producing plants with mutations in six amino acids (mtPgPAP, mtBaPAP, mtMzPAP) (mean ± s.d., ^**^P < 0.01 by Student’s *t*-test, *n* = 3). **c** Activation of *SoANS promoter* by PAPs of betalain-producing plants with mutations in six amino acids (mtPgPAP, mtBaPAP, and mtMzPAP) (mean ± s.d., ^*^P < 0.05 by Student’s *t*-test, *n* = 3)

To determine whether PgPAP can regulate late genes in anthocyanin synthesis, transient expression experiments were conducted in Arabidopsis leaf cells (Fig. 8). We co-bombarded effector constructs containing *PgPAP* with *AtTT8* and *AtTTG1*, which are driven by the *CaMV 35 S* promoter and the promoter regions of *AtANS*, fused in-frame to the *LUC* gene. The transient transformation of Arabidopsis leaf cells with *PgPAP*, *AtTT8*, and *AtTTG1* induced no significant activation of the reporter gene. The alignment of predicted amino acid sequences of PAP homologs of betalain-producing Caryophyllales and anthocyanin-producing plants confirmed that six amino acids (G4, L47, L66, R69, L74, and G83) that are common in PAPs of anthocyanin-producing plants were replaced in betalain-producing Caryophyllales PAPs (V4, F47, H66, K/E69, Y/N74, and S83) (Fig. 7a) isolated in this study. To determine whether the six residues are important for the transactivation ability of PAP, each single-nucleotide base of the codon encoding six residues in PgPAP was changed to the corresponding nucleotide in anthocyanin-producing PAPs (Fig. 7a). The resulting mutated *PgPAP1–6* (V4G, F47L, H66L, K69R, Y74L, and S83G) was fused with the *CaMV 35 S* promoter and used as an effector construct expressing the mutated *PgPAP1–6* (*mtPgPAP*) in dual luciferase assays. As shown in Fig. 8a, replacement of the six amino acids enhanced PgPAP activity. The activity of mtPgPAP was lower than that of AtPAP, but reached 108 % of that of PhAN2, the anthocyanin regulator in *Petunia* (Quattrocchio et al. 1999), as also observed in ectopic expression of combinations of MYB, bHLH, and WDRs on *AtANS* activation. As shown in Fig. S6, all single-amino-acid replacements reduced the full activity of mtPgPAP, implying that all six amino acids are required to retain sufficient activity of the mtPgPAP. Further substitutions other than the six amino acids (I29V, L32R, and E62D) common in PAPs of anthocyanin-producing plants and BvMYB1 showed no significant effect on transactivation ability of mtPgPAP. As observed with PgPAP, two other betalain-producing Caryophyllales PAP homologs, BaPAP and MzPAP, exhibited low transactivation activities for the *AtANS* promoter, whereas activity was recovered by replacing the six amino acids (V4G, F47L, H66L, K/E69R, Y/N74L, and S83G) in other PAP homologs of the betalain-producing Caryophyllales (mtBaPAP and mtMzPAP) (Fig. 8b). Recovery of transactivation activities by replacing the six amino acids of betalain-producing Caryophyllales PAP homologs was also observed for the *SoANS* promoter (Fig. 8c).

## Discussion

Caryophyllales plants possess functional *DFR* and *ANS*, as previously demonstrated through *in vitro* experiments using recombinant proteins (Shimada et al. 2004, 2005); expression profiles revealed that *DFR* and *ANS* are expressed only in seeds in *S. oleracea* (Shimada et al. 2005). However, Polturak et al. (2018) reported that multiple genes in the core phenylpropanoid and anthocyanin biosynthetic pathways, including a homolog of *ANS*, were expressed during the course of floral development of *Mirabilis jalapa*, and suggested that the truncation of a homolog of *M. jalapa ANS* may cause the loss of anthocyanins in *M. jalapa*. This mechanism could explain the broad mutual exclusion of anthocyanins and betalains more broadly, or it may be restricted only to *M. jalapa* or a subset of species in the family Nyctaginaceae (Polturak et al. 2018; Timoneda et al. 2019). In the present study, to confirm the function of *SoANS in vivo*, we attempted molecular complementation of the Arabidopsis *ans* mutant line (*tds4*) with *SoANS* and transient overexpression of *SoANS* through shotgun transformation of *Astrophytum myriostigma* yellow tepals, which accumulate the flavonol glucoside. The increased anthocyanin accumulation observed in transgenic plants overexpressing *SoANS* indicated that the ectopic expression of *SoANS* in *tds4* restored the WT phenotype in an Arabidopsis *ans* mutant. (Fig. 2). Transient overexpression of *SoANS* was also examined in the yellow tepals of an *Astrophytum* cactus (Fig. 3). Red cells were observed in *Astrophytum* tepals introduced with *SoDFR*, *SoANS*, and *AN9*, a *Petunia* glutathione S-transferase required for anthocyanin sequestration (Mueller et al. 2000). The change of a red cell to blue following alkali treatment indicates that the color of the transformed cell is produced by anthocyanins (Fig. S1).

The anthocyanin accumulation in *Astrophytum myriostigma* tepals introduced to *SoDFR*, *SoANS*, and *PhAN9* implies that the expression of *SoDFR*, *SoANS*, and *PhAN9* in *Astrophytum myriostigma* yellow tepals shifts the metabolic flow of dihydroflavonol to anthocyanin from flavonol synthesis in *Astrophytum myriostigma* tepals (Figs. 1 and 3). Anthocyanin accumulation was not observed following the introduction of *SoDFR* and *SoANS* without *PhAN9*. Similarly, anthocyanin accumulation was induced by WDRs, bHLH, and MYB with *PhAN9*, but not without *PhAN9* (Fig. 5b, d), which implies that not only the lack of an anthocyanin transport system but also anthocyanin synthesis in *Astrophytum myriostigma* and *PhAN9* may contribute to anthocyanin transport in *Astrophytum myriostigma*. Vacuolar transport is a critical step in anthocyanin pigmentation, as observed in Arabidopsis *tt19* mutants; the lack of anthocyanin pigmentation appears to be caused by a mutation in the *GST* gene family (Kitamura et al. 2004). Our results suggest that GST is not under the control of PAP or a lack or loss of function of GST itself in betalain-producing Caryophyllales. The induction of anthocyanin accumulation by overexpressing betalain-producing Caryophyllales *DFR* and *ANS* with *PhAN9* in Caryophyllales cells implies that the lack of anthocyanin synthesis may be caused by suppression or limited expression of *DFR* and *ANS* in betalain-producing Caryophyllales plants.

The *SoANSproGUS::* assays showed that *SoANSpro* can be activated by Arabidopsis transcription factors (Fig. S2). To determine whether Arabidopsis transcription factors directly regulate *SoANS*, we conducted a dual luciferase assay in Arabidopsis leaf cells using the *SoANSpro::LUC* construct as a reporter and Arabidopsis *MYB/bHLH/WDR* genes driven by the *CaMV* *35 S* promoter as effectors. The *SoANSpro* was activated by AtPAP1/AtTT8/AtTTG1 in Arabidopsis leaves (Fig. 4B). The transactivation of AtPAP1/AtTT8/AtTTG1 for the *SoANSpro* was less than half of that for *AtANSpro*, but more than threefold higher than that of the co-expression of AtTT2, which activates *AtANS* as a proanthocyanidin regulator, with AtTT8 and AtTTG1. The *SoDFRpro* was also activated by AtPAP1/AtTT8/AtTTG1 in Arabidopsis leaves (Fig. 4c). These findings indicate that the *SoANSpro* and *SoDFRpro* preserve their promoter function and are activated by Arabidopsis transcription factors. In contrast, the *SoANSpro* and *SoDFRpro* were hardly or not activated by PhAN2 and PhJAF13, which are anthocyanin regulators in *Petunia* (Shimada et al. 2007). The conservation of combinational interactions between MYBs and bHLHs from different species for anthocyanin regulation has been demonstrated. Transient expression assays of *Zea mays* and *Petunia hybrida* have shown that *Z. mays* C1 and R or *P*. *hydrida* AN2 and JAF13 can activate the promoter of *Z. mays* *CHS* (*c2*) but not that of *P. hybrida* *CHS* (*chsA*). Thus, regulatory anthocyanin genes are conserved between species and divergent evolution of the target gene promoters is responsible for species-specific differences among regulatory networks (Quattrocchio et al. 1998). The diverse transactivation ability of PAP homologs for the promoters of *ANS* and *DFR* of different plant species shown in transient expression assays (Figs. 4 and 8) may be due to species-specific diversity among regulatory networks.

The anthocyanin accumulation induced by overexpression of the Arabidopsis transcription factors PAP1, TT8/EGL3, and TTG1 with PhAN9 in *Astrophytum* may indicate potential for anthocyanin synthesis in betalain-producing Caryophyllales. The fact that Arabidopsis transcription factors can activate *SoDFRpro* and *SoANSpro* implies that the suppression of late anthocyanin biosynthetic genes may be caused by loss of function of the transcription factors involved in their activation in betalain-producing Caryophyllales. We isolated and characterized PgPAP, which encodes an MYB transcription factor with high amino acid sequence similarity to AtPAP1. The expression profiles of *PgPAP* showed that *PgPAP* transcript levels increased with increasing bud size and decreased with flowering (Fig. 6b), which is consistent with the description of *AtPAP1* being involved in floral transition and early flower development, according to a TAIR Microarray Expression search (https://www.arabidopsis.org/servlets/MultiServlet). The sequence homology and phylogenetic analysis of three Caryophyllales MYB proteins imply that *PgPAP*, *BaPAP*, and *MzPAP* are orthologous of *AtPAP1*, which is involved in the transactivation of late anthocyanin biosynthetic genes (Fig. 7). Notably, amino acid residues R and ANDV, which are common in PAPs (Heppel et al. 2013; Lin-Wang et al. 2010) are conserved in the PAP homologs of Caryophyllales species isolated in this study (Fig. 7a). In this context, we assessed the function of betalain-producing Caryophyllales’ PAPs using transient expression assays in plant cells. We observed no significant transactivation of betalain-producing Caryophyllales PAPs on the *AtANS* promoter (Fig. 8b, c). The alignment of predicted amino acid sequences of PAPs in betalain-producing Caryophyllales and anthocyanin-producing plants showed that six common amino acids among PAPs of anthocyanin-producing plants were replaced in the PAPs of betalain-producing Caryophyllales plants (PgPAP, BaPAP, and MzPAP) (Fig. 7a). The replacement of six amino acid residues in the PAPs of betalain-producing Caryophyllales plants to corresponding residues in anthocyanin-regulating MYB factors (V4G, F47L, H66L, K/E69R, Y/N74L, and S83G) enhanced their transactivation abilities (Fig. 8b, c). The transactivation activities of mtPgPAP were lower than those of AtPAP, which nearly reached those of PhAN2, which is an anthocyanin regulator in *Petunia* (Fig. 8a). Single substitutions of each of the six amino acid residues of mutated PgPAP significantly decreased, but did not abolish, its transactivation activity (Fig. S6), implying that all six residues are critical to their functions as transcriptional activators.

The activation of the *ANS* promoter through heterologous expression of the mutated Caryophyllales PAPs in Arabidopsis leaves (mtPgPAP, mtBaPAP, and mtMzPAP) (Fig. 8B, C) implies loss-of-function in betacyanin-producing Caryophyllales PAPs during transactivation of late genes of anthocyanin biosynthesis. Loss-of-function in the MYB transcription factor may suppress the expression of late genes of anthocyanin synthesis, resulting in a lack of anthocyanin in betacyanin-producing Caryophyllales. Caryophyllales anthocyanin regulators have been shown to change their levels of activity through alteration of a few amino acid residues. These substitutions in the PAPs of Caryophyllales plants may represent adaptive evolutionary changes that led to the suppression of gene expression in the anthocyanin biosynthetic pathway. The expression profiles of *PgPAP* (Fig. 6b) implied that Caryophyllales PAPs may contribute to the regulation of gene expression other than anthocyanin biosynthetic pathway during flower development.

BvMYB1, which has been shown to upregulate *BvDOD* during betacyanin synthesis in beets, was unable to interact with bHLH in anthocyanin-producing plants due to the lack of five of seven conserved amino acids that are important for bHLH interaction (Hatlestad et al. 2015; Zimmermann et al. 2004) identified a conserved amino acid signature [D/E]Lx2[R/K]x 3Lx6Lx3R at positions 12–33 in the R3–MYB domain, which contributes to the interaction between MYB and bHLH proteins. In the present study, two of the six amino acid residues replaced in mutated Caryophyllales PAPs corresponded to those of conserved amino acids (Figs. 7a and 8). Each substitution of these two amino acids (H66L and K69R) reduced, but did not abolish, the full activity of mutated PgPAP1 (Fig S6). Through alteration of a few amino acid residues, PAPs of betacyanin-producing Caryophyllales may interact with an unknown bHLH involved in the transcriptional activation of genes in another biosynthetic pathway. Changes in the target genes of transactivation via alternation of the partnership between MYB and bHLH could create a new biosynthetic pathway that would contribute to the development of a diverse array of secondary metabolites in higher plants.

### Data deposition

The nucleotide sequences reported in this paper have been submitted to DDBJ under accession numbers MW192234 (*PgPAP*), MW192235 (*MzPAP*), MW192236 (*BaPAP*) and MW192237 (*PgPAP genome*).

We thank Dr Ronald E Koes (University of Amsterdam) for the gift of the AN2 cDNA, Dr Yasuo Niwa (University of Shizuoka) for the kind gift of the 35 S::sGFP (S65T) plasmid, Dr. Tsuyoshi Nakagawa (Shimane University) for the gift of the gateway vector, Dr Kazuki Saito (Chiba University) for the gift of the *PfANS* clone and Eri Wakui, Maki Yamamoto, Saki Maegawa, Akane Kasei for technical assistance.

## Supplementary Information

Below is the link to the electronic supplementary material.

Supplementary material 1 (PDF 585.2 kb)

## References

[CR1] Baudry A, Caboche M, Lepiniec L (2006). TT8 controls its own expression in a feedback regulation involving TTG1 and homologous MYB and bHLH factors, allowing a strong and cell-specific accumulation of flavonoids in *Arabidopsis thaliana*. Plant J.

[CR2] Baudry A, Heim MA, Dubreucq B, Caboche M, Weisshaar B, Lepiniec L (2004). TT2, TT8, and TTG1 synergistically specify the expression of BANYULS and proanthocyanidin biosynthesis in *Arabidopsis thaliana*. Plant J.

[CR3] Brockington SF, Walker RH, Glover BJ, Soltis PS, Soltis DE (2011). Complex pigment evolution in the Caryophyllales. New Phytol.

[CR4] Broun P (2005). Transcriptional control of flavonoid biosynthesis: a complex network of conserved regulators involved in multiple aspects of differentiation in Arabidopsis. Curr Opin Plant Biol.

[CR5] Clement JS, Mabry TJ (1996). Pigment evolution in the caryophyllales: a systematic overview. Botanica Acta.

[CR6] Constabel CP (2018). Molecular controls of proanthocyanidin synthesis and structure: prospects for genetic engineering in crop plants. J Agric Food Chem.

[CR7] Forkmann G, Martens S (2001). Metabolic engineering and applications of flavonoids. Curr Opin Biotechnol.

[CR8] Freixas Coutin JA, Munholland S, Silva A, Subedi S, Lukens L, Crosby WL (2017). Proanthocyanidin accumulation and transcriptional responses in the seed coat of cranberry beans (*Phaseolus vulgaris* L.) with different susceptibility to postharvest darkening. BMC Plant Biol.

[CR9] Gonzalez A, Zhao M, Leavitt JM, Lloyd AM (2007). Regulation of the anthocyanin biosynthetic pathway by the TTG1/bHLH/Myb transcriptional complex in Arabidopsis seedlings. Plant J.

[CR10] Grotewold E, Sainz MB, Tagliani L, Hernandez JM, Bowen B, Chandler VL (2000). Identification of the residues in the Myb domain of maize C1 that specify the interaction with the bHLH cofactor R. Proc Natl Acad Sci USA.

[CR11] Grützner R, Schubert R, Horn C, Yang C, Vogt T, Marillonnet S (2021). Engineering betalain biosynthesis in tomato for high level betanin production in fruits. Front Plant Sci.

[CR12] Hatlestad GJ, Akhavan NA, Sunnadeniya RM, Elam L, Cargile S, Hembd A (2015). The beet Y locus encodes an anthocyanin MYB-like protein that activates the betalain red pigment pathway. Nature Genet.

[CR13] Hatlestad GJ, Sunnadeniya RM, Akhavan NA, Gonzalez A, Goldman IL, McGrath JM (2012). The beet R locus encodes a new cytochrome P450 required for red betalain production. Nature Genet.

[CR14] Heppel SC, Jaffe FW, Takos AM, Schellmann S, Rausch T, Walker AR (2013). Identification of key amino acids for the evolution of promoter target specificity of anthocyanin and proanthocyanidin regulating MYB factors. Plant Mol Biol.

[CR15] Hirano H, Sakuta M, Komamine A (1992). Inhibition by cytokinin of the accumulation of betacyanin in suspension cultures of *Phytolacca americana*. Z Naturforsch.

[CR16] Hirano H, Sakuta M, Komamine A (1996). Inhibition of betacyanin accumulation by abscisic acid in suspension cultures of *Phytolacca americana*. Z Naturforsch.

[CR17] Holton TA, Cornish EC (1995). Genetics and biochemistry of anthocyanin biosynthesis. Plant Cell.

[CR18] Ito J, Fukuda H (2002). ZEN1 is a key enzyme in the degradation of nuclear DNA during programmed cell death of tracheary elements. Plant Cell.

[CR19] Iwashina T (2001). Flavonoids and their distribution in plant families containing the betalain pigments. Ann Tsukuba Bot Gard.

[CR20] Iwashina T, Ootani S, Hayashi K (1988). On the pigmentation spherical bodies and crystals in tepals of Cactaceous species in reference to the nature of betalains or flavonols. Bot Mag Tokyo.

[CR21] Jain G, Gould KS (2015). Are betalain pigments the functional homologues of anthocyanins in plants?. Environ Exp Bot.

[CR22] Jun J, Xiao X, Rao X, Dixon RA (2018). Proanthocyanidin subunit composition determined by functionally diverged dioxygenases. Nat Plants.

[CR23] Kitamura S, Shikazono N, Tanaka A (2004). TRANSPARENT TESTA 19 is involved in the accumulation of both anthocyanins and proanthocyanidins in Arabidopsis. Plant J.

[CR24] Koes R, Verweij W, Quattrocchio F (2005). Flavonoids: a colorful model for the regulation and evolution of biochemical pathways. Trends Plant Sci.

[CR25] Lin-Wang K, Bolitho K, Grafton K, Kortstee A, Karunairetnam S, McGhie TK (2010). An R2R3 MYB transcription factor associated with regulation of the anthocyanin biosynthetic pathway in Rosaceae. BMC Plant Biol.

[CR26] Lloyd A, Brockman A, Aguirre L, Campbell A, Bean A, Cantero A (2017). Advances in the MYB–bHLH–WD Repeat (MBW) pigment regulatory model: addition of a WRKY factor and co-option of an anthocyanin MYB for betalain regulation. Plant Cell Physiol.

[CR27] Lopez-Nieves S, Yang Y, Timoneda A, Wang M, Feng T, Smith SA (2018). Relaxation of tyrosine pathway regulation underlies the evolution of betalain pigmentation in Caryophyllales. New Phytol.

[CR28] Mabry TJ, Bell EA, Charlwood BV (1980). Betalains. Secondary plant products. Encyclopedia of plant physiology.

[CR29] Mueller LA, Goodman CD, Silady RA, Walbot V (2000). AN9, a petunia glutathione S-transferase required for anthocyanin sequestration, is a flavonoid-binding protein. Plant Physiol.

[CR30] Niu N, Xu C, Zhang W, Zhang B, Li X, Lin-Wang K (2010). Coordinated regulation of anthocyanin biosynthesis in Chinese bayberry (*Myrica rubra*) fruit by a R2R3 MYB transcription factor. Planta.

[CR31] Park KI, Nitasaka E, Hoshino A (2018). Anthocyanin mutants of Japanese and common morning glories exhibit normal proanthocyanidin accumulation in seed coats. Plant Biotechnol.

[CR32] Pattanaik S, Kong Q, Zaitlin D, Werkman JR, Xie CH, Patra B (2010). Isolation and functional characterization of a floral tissue-specific R2R3 MYB regulator from tobacco. Planta.

[CR33] Polturak G, Heinig U, Grossman N, Battat M, Leshkowitz D, Malitsky S (2018). Transcriptome and metabolic profiling provides insights into betalain biosynthesis and evolution in *Mirabilis jalapa*. Mol Plant.

[CR34] Quattrocchio F, Wing JF, van der Woude K, Mol J, Koes R (1998). Analysis of bHLH and MYB domain proteins: species specific regulatory differences are caused by divergent evolution of target anthocyanin genes. Plant J.

[CR35] Quattrocchio F, Wing J, van der Woude K, Souer E, de Vetten N (1999). Molecular analysis of the anthocyanin2 gene of petunia and its role in the evolution of flower color. Plant Cell.

[CR36] Ramakrishna A, Ravishankar GA (2011). Influence of abiotic stress signals on secondary metabolites in plants. Plant Signal Behav.

[CR37] Ramsay NA, Glover BJ (2005). MYB–bHLH–WD40 protein complex and the evolution of cellular diversity. Trends Plant Sci.

[CR38] Saito K, Kobayashi M, Gong Z, Tanaka Y, Yamazaki M (1999). Direct evidence for anthocyanidin synthase as a 2-oxoglutarate-dependent oxygenase: molecular cloning and functional expression of cDNA from a red forma of *Perilla frutescens*. Plant J.

[CR39] Saitou N, Nei M (1987). The neighbor-joining method: a new method for reconstructing phylogenetic trees. Mol Biol Evol.

[CR40] Sakuta M, Hirano H, Komamine A (1991). Stimulation by 2,4-dichlorophenoxyacetic acid of betacyanin accumulation in suspension cultures of *Phytolacca americana*. Physiol Plant.

[CR41] Sheehan H, Feng T, Walker-Hale N, Lopez-Nieves S, Pucker B, Guo R (2020). Evolution of l-DOPA 4,5-dioxygenase activity allows for recurrent specialisation to betalain pigmentation in Caryophyllales. New Phytol.

[CR42] Shimada S, Inoue YT, Sakuta M (2005). Anthocyanidin synthase in non-anthocyanin-producing Caryophyllales species. Plant J.

[CR43] Shimada S, Otsuki H, Sakuta M (2007). Transcriptional control of anthocyanin biosynthetic genes in the Caryophyllales. J Exp Bot.

[CR44] Shimada S, Takahashi K, Sato Y, Sakuta M (2004). Dihydroflavonol 4-reductasecDNA from non-Anthocyanin-Producing Species in the Caryophyllales. Plant Cell Physiol.

[CR45] Solfanelli C, Poggi A, Loreti E, Alpi A, Perata P (2006). Sucrose-specifica induction of the anthocyanin biosynthetic pathway in Arabidopsis. Plant Physiol.

[CR46] Stafford HA (1994). Anthocyanins and betalains: evolution of the mutually exclusive pathways. Plant Sci.

[CR47] Takahashi K, Takamura E, Sakuta M (2009). Isolation and expression analysis of two DOPA dioxygenases in *Phytolacca americana*. Z Natureforsch.

[CR48] Thulin M, Moore AJ, El-Seedi H, Larsson A, Christin P-A, Edwards EJ (2016). Phylogeny and generic delimitation in Molluginaceae, new pigment data in Caryophyllales, and the new family Corbichoniaceae. TAXON.

[CR49] Timoneda A, Sheehan H, Feng T, Lopez-Nieves S, Maeda HA, Brockington SF (2018). Redirecting primary metabolism to boost production of tyrosine-derived specialised metabolites *in Planta*. Sci Rep.

[CR50] Timoneda A, Feng T, Sheehan H, Walker-Hale N, Pucker B, Lopez-Nieves S (2019). The evolution of betalain biosynthesis in Caryophyllales. New Phytol.

[CR51] Venkataraman K, Geissman TA (1962). Methods for determining the structure of flavonoid compounds. The chemistry of flavonoid compounds.

[CR52] Winkel-Shirley B (2001). Flavonoid biosynthesis. A colorful model for genetics, biochemistry, cell biology, and biotechnology. Plant Physiol.

[CR53] Yoshida K, Iwasaka R, Kaneko T, Sato S, Tabata S, Sakuta M (2008). Functional differentiation of *Lotus japonicus* TT2s, R2R3-MYB transcription factors comprising a multigene family. Plant Cell Physiol.

[CR54] Yoshida K, Iwasaka R, Shimada N, Ayabe S, Aoki T, Sakuta M (2010). Transcriptional control of the dihydroflavonol 4-reductase multigene family in *Lotus japonicas*. J Plant Res.

[CR55] Zimmermann IM, Heim MA, Weisshaar B, Uhrig JF (2004). Comprehensive identification of *Arabidopsis thaliana* MYB transcription factors interacting with R/B-like BHLH proteins. Plant J.

