# Correction: Anthocyanin synthesis potential in betalain-producing Caryophyllales plants

**DOI:** 10.1007/s10265-022-01386-9

**Published:** 2022-03-18

**Authors:** Masaaki Sakuta, Asuka Tanaka, Kaori Iwase, Mizuki Miyasaka, Sachiko Ichiki, Miho Hatai, Yoriko T. Inoue, Ayumi Yamagami, Takeshi Nakano, Kazuko Yoshida, Setsuko Shimada

**Affiliations:** 1 Department of Biological Sciences, Ochanomizu University, 112-8610 Tokyo, Japan; 2 Graduate School of Biostudies, Kyoto University, 606-8502 Kyoto, Japan; 3 Present Address: Organization for the Strategic Coordination of Research and Intellectual Properties, Meiji University, 214-8571 Kanagawa, Japan; 4 Present Address: Synthetic Genomics Research group, RIKEN Center for Sustainable Resource Science, 230-0045 Yokohama, Kanagawa Japan

## Correction to: Journal of Plant Research (2021) 134:1335–1349 10.1007/s10265-021-01341-0

The article “Anthocyanin synthesis potential in betalain-producing Caryophyllales plants”, written by Masaaki Sakuta, Asuka Tanaka, Kaori Iwase, Mizuki Miyasaka, Sachiko Ichiki, Miho Hatai, Yoriko T. Inoue, Ayumi Yamagami, Takeshi Nakano, Kazuko Yoshida, Setsuko Shimada, was originally published Online First without Open Access. After publication in volume 134, issue 6, page 1335–1349 the author decided to opt for Open Choice and to make the article an Open Access publication. Therefore, the copyright of the article has been changed to © The Author(s) 2022 and the article is forthwith distributed under a Creative Commons Attribution 4.0 International License, which permits use, sharing, adaptation, distribution and reproduction in any medium or format, as long as you give appropriate credit to the original author(s) and the source, provide a link to the Creative Commons licence, and indicate if changes were made. The images or other third party material in this article are included in the article’s Creative Commons licence, unless indicated otherwise in a credit line to the material. If material is not included in the article’s Creative Commons licence and your intended use is not permitted by statutory regulation or exceeds the permitted use, you will need to obtain permission directly from the copyright holder. To view a copy of this licence, visit http://creativecommons.org/licenses/by/4.0/.

