# Supplementary material for: Anthocyanin synthesis potential in betalain-producing Caryophyllales plants
Source: J Plant Res. 2021 Sep 3;134(6):1335–49. doi: 10.1007/s10265-021-01341-0 (PMC8930957; doi:10.1007/s10265-021-01341-0)
Supplement: Supplementary file 1 — Supplementary material 1 (PDF 585.2 kb) [file 10265_2021_1341_MOESM1_ESM.pdf]

Supplementary Materials

Journal of Plant Research

Anthocyanin synthesis potential in betalain-producing Caryophyllales plants

Masaaki Sakuta[1,3], Asuka Tanaka[1], Kaori Iwase[1], Mizuki Miyasaka[1], Sachiko Ichiki[1], Miho Hatai[1], Yoriko T. Inoue[1], Ayumi Yamagami[2], Takeshi Nakano[2], Kazuko Yoshida[1] and Setsuko Shimada[1, 4]

[1]Department of Biological Sciences, Ochanomizu University, Tokyo, 112-8610 Japan
[2]Graduate School of Biostudies, Kyoto University, Kyoto, 606-8502, Japan
[3]Present address: Organization for the Strategic Coordination of Research and Intellectual Properties, Meiji University, Kawasaki, Kanagawa 214-8571, Japan
[4]Present address: Synthetic Genomics Research group, RIKEN Center for Sustainable Resource Science, Yokohama, Kanagawa 230-0045, Japan

sakuta.masaaki@ocha.ac.jp

Table S1. Primer sequences used in this study

**Genotyping**	
ANS-GT-L	GATTGTTTTACTAAAGAAAACAAA
ANS-GT-R	GTTAAAGCGCTTACATCGGTG
**Complementation analysis**	
spiANS-gw-F	CACCATGACGGCCACAATTGCTC
spiANS-gw-R	TTATGGATCTTGAGCTTCC
perANS-gw-F	CACCATGGTTACGCGTGCAATGGG
perANS-gw-R	CTAGTACGTTGGCTTCTCAT
ANS-F1	CGCGAAGTGTCTTCGTTTG
ANS-R3	GAAAAGCTGCAAACCCGGAA
spiA	TAGTCAGCAGGAGTCTTAGG
perANS-F	GATTTTATCAGTCCTATCAATCGGG
Actin-F1	GTGAAGGCTGGATTTGCAGGA
Actin-R1	AACCACCGATCCAGGCACTGT
**Promoter-GUS transgenic lines**	
SoANSs2	GCAAGCTTAGAGATATGATAGAGAT
SoANSas2	GAAGAAATATGGCTTGACTAAAGC
**Transient expression assay**	
AtPAP-S-Sal I	ATGCGTCGACATGGAGGGTTCGTCCAAAGG
AtPAP-AS-Not I	GTCAGCGGCCGCTCTAATCAAATTTCACAGT
**Isolation of PAP homologs from the Caryophyllales**	
PAPlikesearch1F	GG(A/G)GTGAG(A/G)AAAGGTGCATGGAC
PAPlikesearch1R	CCAGTA(A/G)TT(C/T)TTGACATCATT
PAPlikesearch2F	AA(A/G)AG(C/T)TG(C/T)AGATT(A/G)AG(A/G)TGG
PAPlikesearch2R	TTGACATCATTAGCGGTCC(G/T)(A/T)CC
TAIL-PCR/AD2	NGTCGA(G/C)(A/T)GANA(A/T)GAA
TAIL-PCR/AD5	(G/C) (G/C)TGG(G/C)TANAT(A/T)AT(A/T)CT
PgPAP5'TAIL1	CCCGGATGCCTACTAGCTATGAGTG
PgPAP5'TAIL2	GGATCAACACCCAAGGTAGGCAGG
PgPAP5'TAIL3	GGAGGAAACTAGCTAGCTAGTGTG
PgPAP3'TAIL1	GGGAGCTTCACCAATGAGGAGGTGG
PgPAP3'TAIL2	CACACTAGCTAGCTAGTTTCCTCC
PgPAP3'TAIL3	CCTGCCTACCTTGGGTGTTGATCC
PgPAP5'TAIL4	CCACCTCCTCATTGGTGAAGCTCCC
PgPAP5'TAIL5	GTTGTTAGAACGGATTTAATTTGCC
PgPAP3'TAIL4	GTAGGTGGTGTTTCTATCACTACTG
PgPAP5'TAIL6	GTTTGATGATGTGTTCCACCTCC
PgPAP3'TAIL5	GGAGGTGGAACACATCATCAAAC
PgPAP5'TAIL7	GAGCTTGTGAAGTTTGATGATGTG
PgPAP5'TAIL8	CATGCAACATGGGAACTTTGGAG
**3', 5'-RACE**	
ODTA	CACGCGTATCGATGTTTTTTTTTTTTTTTTTTTT
AUAP(dG)	GGCCACGCGTCGACTAGTACGGGGGGGGGGGGGG
BaPAPlike3'RACE 1	GATTGCTAGTAGGCTACCAGGGA
BaPAPlike3'RACE 2	CAGGGAGAACAGCAAATGATG
BaPAPlike5'RACE 1	GACCACCTATTGCCGTTGAG
BaPAPlike5'RACE 2	CGATGATATGTTCGACCTCCTC

BaPAP-F-SalI	ATGCGTCGACATGGGAGGAGTTGCATG
BaPAP-R-NotI	GTCAGCGGCCGCTCACAAAGACTGGG
MzPAPlike3'RACE 1	AAGAGTTGTAGATTGAGGTGGTT
MzPAPlike3'RACE 2	GGCAATAGGTGGTCACTCATAGCT
MzPAPlike5'RACE 1	GACCACCTATTGCCATAGAG
MzPAPlike5'RACE 2	TGATGATGTGTTCAACTTCCTC
MzPAP-F-SalI	ATGCGTCGACATGGGAGGAGTTGCATGGAC
MzPAP-R-NotI	GTCAGCGGCCGCTCAGAAAGAATCAGTCCATAG

RT-PCR

PgPAP-ACTIN-F	TCGAGACATTCAATGTCCCTGC
PgPAP-ACTIN-R	ACCTCTCAGCTCCGATAGTGAT
RT-PCR_PgPAP-F	GAGGAGTTGCCTGGACTGAA
RT-PCR_PgPAP-R	TCACAGAGAGTTAGTCCACAATTCC

Point mutation

V4GF	GAGGTGCCTGGACTGAAGAGGAA
V4GR	CTCCCATGTCGACTGTAATTG
F47LF	GGTTGAACTATTTACGTCCTAAC
F47LR	ATCTCAATCTGCAACTTTTTCGACAC
H66LF	CTCATCATCAAACTTCACAAGCTC
H66LR	TTCCACCTCCTCATTGGTGAA
K69RF	CATCAGGCTTCACAAGCTCTATGG
K69RR	ATGTGTTCCACCTCCTCATTGG
Y74LF	GCTCTTAGGAAATAGGTGGTCAC
Y74LR	TTGTGAAGTTTGATGATGTGTTCC
S83GF	GGTAGGCTACCGGGGAGGACAG
S83GR	AGCTATGAGTGACCACCTATTTCC

Table S2. List of genes in phylogenetic analysis

LjTT2a	BAG12893
LjTT2b	BAG12894
LjTT2c	BAG12895
AtTT2	Q9FJA2
ZmC1	AAA33482
ZmPL	AAB67721
PmMBF1	AAA82943
AmMIXTA	CAA55725
PhMYB1	CAA78386
AtMYB12	AAC83586
ZmP	AAC49394
AtGL1	BAA86879
AtWER	Q9SEI0
VvMYB5A	AAS68190
PhPH4	AAY51377
AtPAP1	AAG42001
AtPAP2	AAG42002
VvMYBA1	BAD18977
LeAN1	AAQ55181
PhAN2	AAF66727
BvMYB1	AET43457
PgPAP	MW192234
MzPAP	MW192235
BaPAP	MW192236

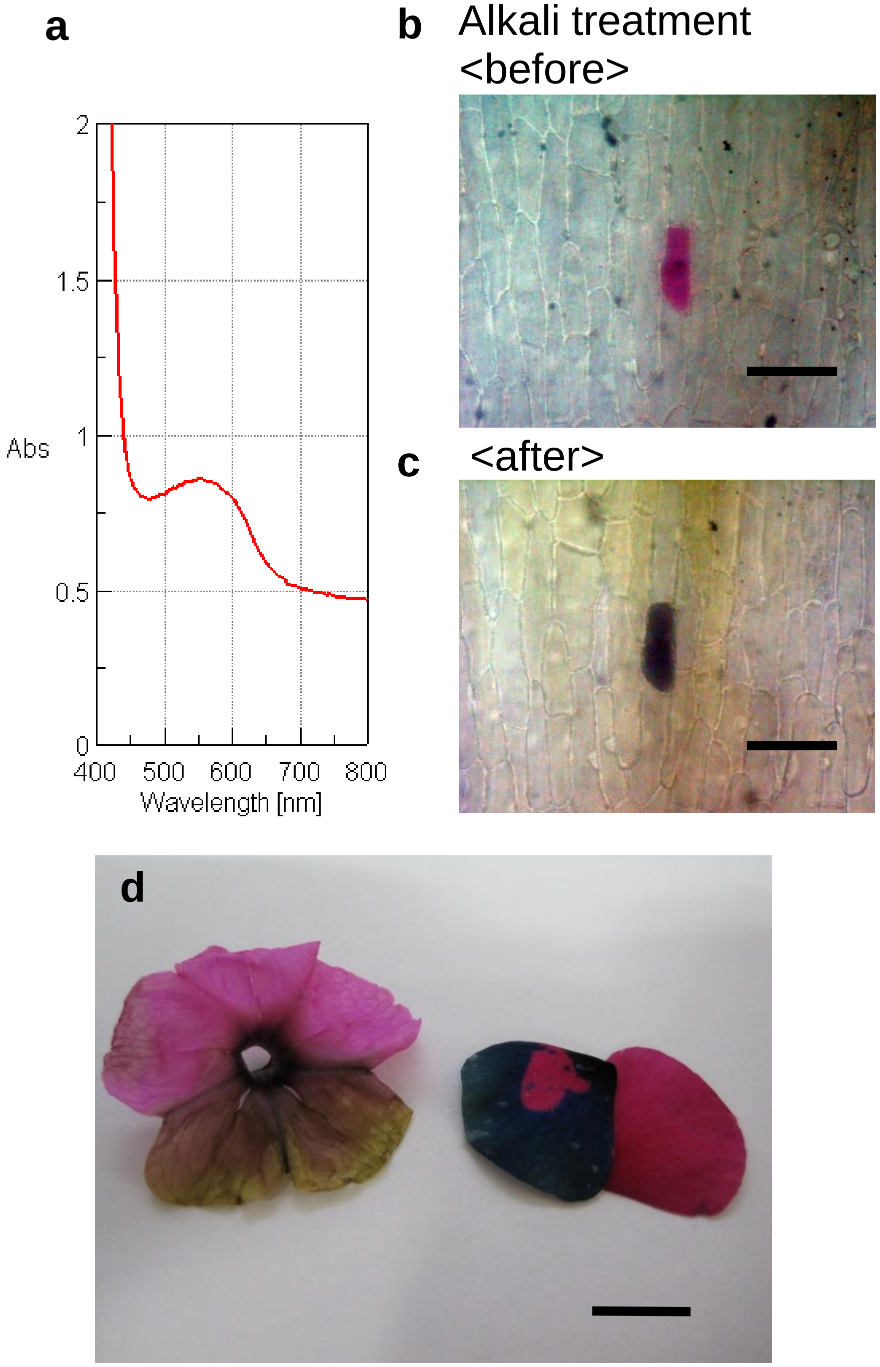

Fig. S1 **a** The absorption spectra of red cells were measured in the visible region (400–800 nm). Colors of transformed cells before (**b**) and after (**c**) alkali treatment with 25% ammonia water. Bars represent 50 µm. **d** Color change showing betacyanin accumulation in *Mirabilis jalapa* petals (left) and anthocyanin accumulation in *Rosa* sp. (right). Some petals were treated with 25% ammonia water. The bar represents 10 mm.

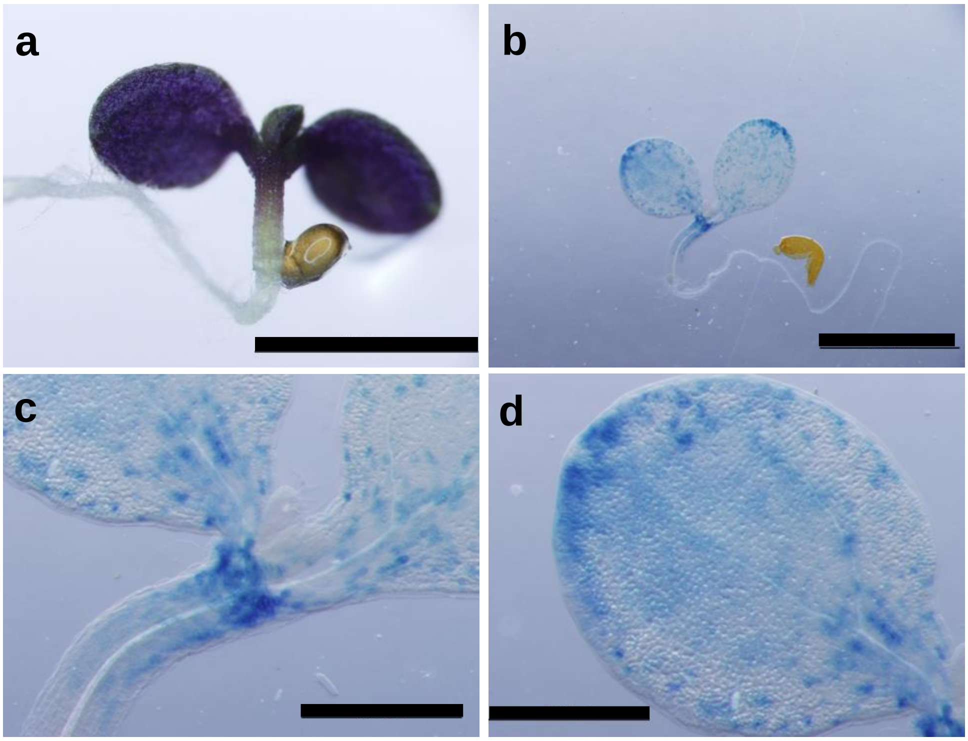

Fig. S2 **a** Anthocyanin-accumulating seedlings at 3 days after germination on 1/2 MS agar medium containing 5% sucrose. *SoANS promoter*::GUS expression pattern in (**b**) whole seedlings, (**c**) basilar cotyledons, and (**d**) cotyledons. Bars in **a** and **b** represent 2.0 mm. Bars in **c** and **d** represent 500 μm.

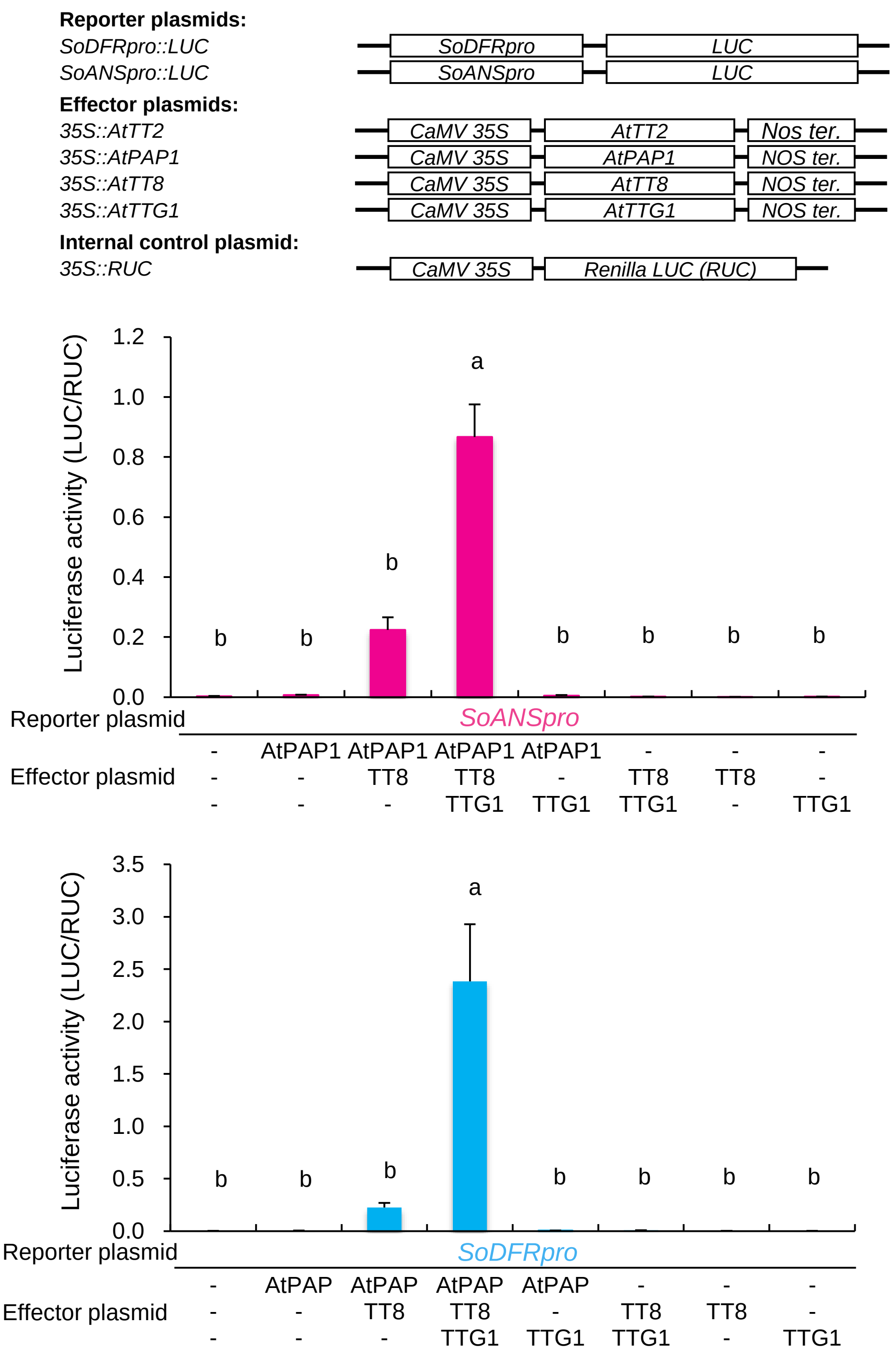

Fig. S3 Transient expression of the reporter constructs of *SoANSpro* and *SoDFRpro*, which contain *LUC* as a reporter gene, with or without expression of *Arabidopsis* transcription factors PAP1/TT8/TTG1 in particle-bombarded *Arabidopsis* leaves. Reporter gene activity, which was measured in the LUC enzyme, is expressed in arbitrary units after normalization to RUC activity expressed from a co-bombarded inner control plasmid, *35S-RUC* (mean ± s.d, $P < 0.05$ by Tukey's test, n = 3). The reporter and effector plasmid constructs (described in Materials and Methods) are shown in the upper part of the figure.

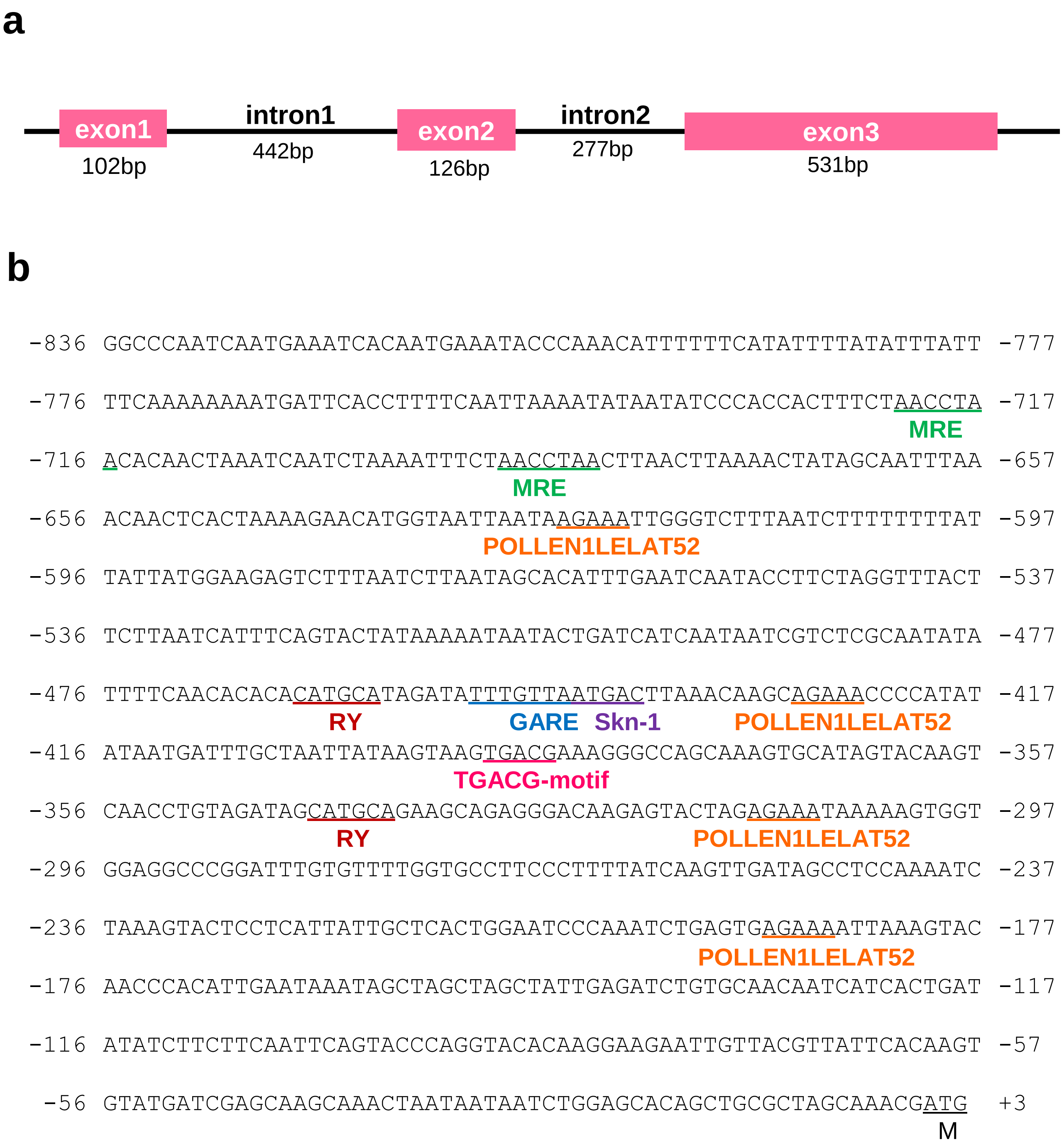

Fig. S4 PgPAP genome sequence **a** Genomic organization of the PgPAP gene (GenBank accession No. MW192237). Numbers under the lines and boxes indicate the number of base pairs in each intron and exon, respectively. **b** Sequences of promoter of *P. grandiflora* ANS. The putative translation initiation codon (ATG) is underlined. In the promoter, four POLLEN1LELAT52, which is involved in pollen-specific transcription; two MREs, which are involved in light regulation; and two RY motifs, which are conserved in seed specific promoters, were found. Skn-1, which is involved in endosperm formation; a gibberellin responsive element (GARE); and a methyl jasmonate responsive motif (TCAGC), were found.

```
AtPAP1     ------MEGSSKGLRKGAWTTEEDSLLRQCINKYGEGKWHQVPVRAGLNRCRKSCRLRWLNYLKPSIKRG 64
PhAN2      ---MSTSNASTSGVRKGAWTEEEDLLLRECIDKYGEGKWHLVPVRAGLNRCRKSCRLRWLNYLRPHIKRG 67
VvMYBA1    --------MESLGVRKGAWIQEEDVLLRKCIEKYGEGKWHLVPLRAGLNRCRKSCRLRWLNYLKPDIKRG 62
PgPAP      -------------MGGVAWTEEEDRLLRECIQRYGEGKWHRIPLLAGLNRCRKSCRLRWFNYLRPNIKRG 57
BaPAP      -------------MGGVAWTEEEDRLLRECIQRYGEGKWHRIPLLAGLNRCRKSCRLRWFNYLRPNIKRG 57
MzPAP      -------------MGGVAWTEEEDRLLRECIHRYGEGKWHRIPLLAGLNRCRKSCRLRWFNYLRPNIKRG 57
BvMYB1     MYQQNSETGSLGRVVKGSWSDEEDDLLRKCIQKYGEGNWKRVPERAGLNRCRKSCRWRWLNYLKPSIKRG 70
AtTT2      MGKRATTSVRREELNRGAWTDHEDKILRDYITTHGEGKWSTLPNQAGLKRCGKSCRLRWKNYLRPGIKRG 70
VvMYBPA1   MGR--APCCSKVGLHRGSWTAREDTLLTKYIQAHGEGHWRSLPKKAGLLRCGKSCRLRWMNYLRPDIKRG 68

AtPAP1     KLSSDEVDLLLRLHRLLGNRWSLIAGRLPGRTANDVKNYWNTHLSKKHEPCCKIKMKKRDIT-PI--PTT 131
PhAN2      DFSLDEVDLILRLHKLLGNRWSLIAGRLPGRTANDVKNYWNTHLRKKLIAPHDQKQESKNK-------AV 130
VvMYBA1    EFALDEVDLMIRLHNLLGNRWSLIAGRLPGRTANDVKNYWHSHHFKKEVQFQEEGRDKPQTHSKT--KAI 130
PgPAP      SFTNEEVEHIIKLHKLYGNRWSLIASRLPGRTANDVKNYWNCHLSKRLLNNAHLIEPNETTKNT---TII 124
BaPAP      NFTNEEVEHIIELHKLNGNRWSLIASRLPGRTANDVKNYWNCHLSKR-LNNSYQTESNQKTGLP---QDE 123
MzPAP      SFTSEEVEHIIKLHKLYGNRWSLIASRLPGRTANDVKNYWNCHLSKR-LNNPHHVEPKEITNN----SVH 122
BvMYB1     HFNEDEVKFIIQQHKLLGNRWSLIAAKLPGRTINDVKNYCNTHLYKKHSIEN-IPAPATNT-------MT 132
AtTT2      NISSDEEELIIRLHNLLGNRWSLIAGRLPGRTDNEIKNHWNSNLRKRL------PKTQTKQ-----PKRI 129
VvMYBPA1   NITPDEDDLIIRLHSLLGNRWSLIAGRLPGRTDNEIKNYWNTHLSKKLRSQGTDPNTHKKMTEPPEPKRR 138

AtPAP1     PALKNNVYK-------------PRPRSFTVNNDCNHLNAPPKVDVNPPCLGLNINNVCDNSIIYNKDKKK 188
PhAN2      KITENNIIK-------------PRPRTFSR-PAMNNFPCWNGKSCNKNTIDKNEG---DTEIIKFSDEK- 182
VvMYBA1    KPHPHKFSK-------------ALPRFELKTTAVDTFDTQVSTSRKPSSTSPQPNDDIIWWESLLAEHAQ 187
PgPAP      AQDKNNTIE-------------CESYEYNWKRSSETIQNNNNPIETISMHDPKPNQASNGGTLLPIEIMS 181
BaPAP      IN--NNLIK-------------CESHEY-WLGQ------GNDPVEFNPMN----------------EIS- 154
MzPAP      VQDKNNNIQ-------------CESHEY-WKRS-EIIQTNN-PVLFSPMHEPRPTQGST----IPKEII- 171
BvMYB1     HNTSSCVDR-------------PESS-----ATIKEPKWWENILVELQREE-KEGKSQNCSGLDFEQDNL 183
AtTT2      KHSTNNENN------VCVIRTKAIRCSKTLLFSDLSLQKKSSTSP-LPLKEQEMDQG-GSSLMGDLEFDF 191
VvMYBPA1   KNTRTRTNNGGGSKRVKISKDQENSNHKVHLPKPVRVTSLISMSRNNSFESNTVSGGSGSSSGGNGETLP 208

AtPAP1     DQLVNNLIDGDNMWLEKFLEESQEVDILVPEATTTEKGDTLAFDVDQLWSLFDG-------ETVKFD--- 248
PhAN2      -QKPEESIDDGLQWWANLLANNIEIEELVSCNSPTLLHEETAPSVNAESSLTQGGGSGLSDFSVDIDDIW 251
VvMYBA1    MDQETDFSASGEMLIASLRTEETATQKKGPMDGMIEQIQGGEGDFPFDVGFWDT-------PNTQVNHLI 250
PgPAP      PFVRDEAYNDQMVA--QEYNKYACIEENGTMEVLQRDLDFELEEIKITTGEHSKCKWDFDELAFDLELWT 249
BaPAP      PYVQAESCDDQAVHKHEEYD-YSCAEEQGIVEELPKDMGFQLDG---------NFKWDFDDLAFDVELWT 214
MzPAP      PYVRDESYDDQTALNQEYYEKYLGAEEHGSVEELSKGMDFELEEIKITMGEQSKFKWDFDELAFDLELWT 241
BvMYB1     GQQDPNINDGMDQWLNSLKE--------VPNLSYQWEENLLDFDVVNLWA-------------------- 225
AtTT2      DRIHSEFHFPDLMDFDGLD--CGN--VTSLVSSN--EILGELVPAQGNLD-LNRPFTSCHHRGDDEDWLR 254
VvMYBPA1   WPSFRDIRDDKVIGVDGVDFFIGDDQGQDLVASSDPESQSHMPPTDNSLEKLYEEYLQLLEREDTQVQLD 278

AtPAP1     -------- 248
PhAN2      DLVS---- 255
VvMYBA1    -------- 250
PgPAP      NSL----- 252
BaPAP      QSL----- 217
MzPAP      DSF----- 244
BvMYB1     -------- 225
AtTT2      DFTC---- 258
VvMYBPA1   SFAESLLI 286
```

Fig. S5 Comparison of the amino acid sequences of PAP and TT2 homorogs. Accession numbers of genes are listed in Supplementary Table S2.

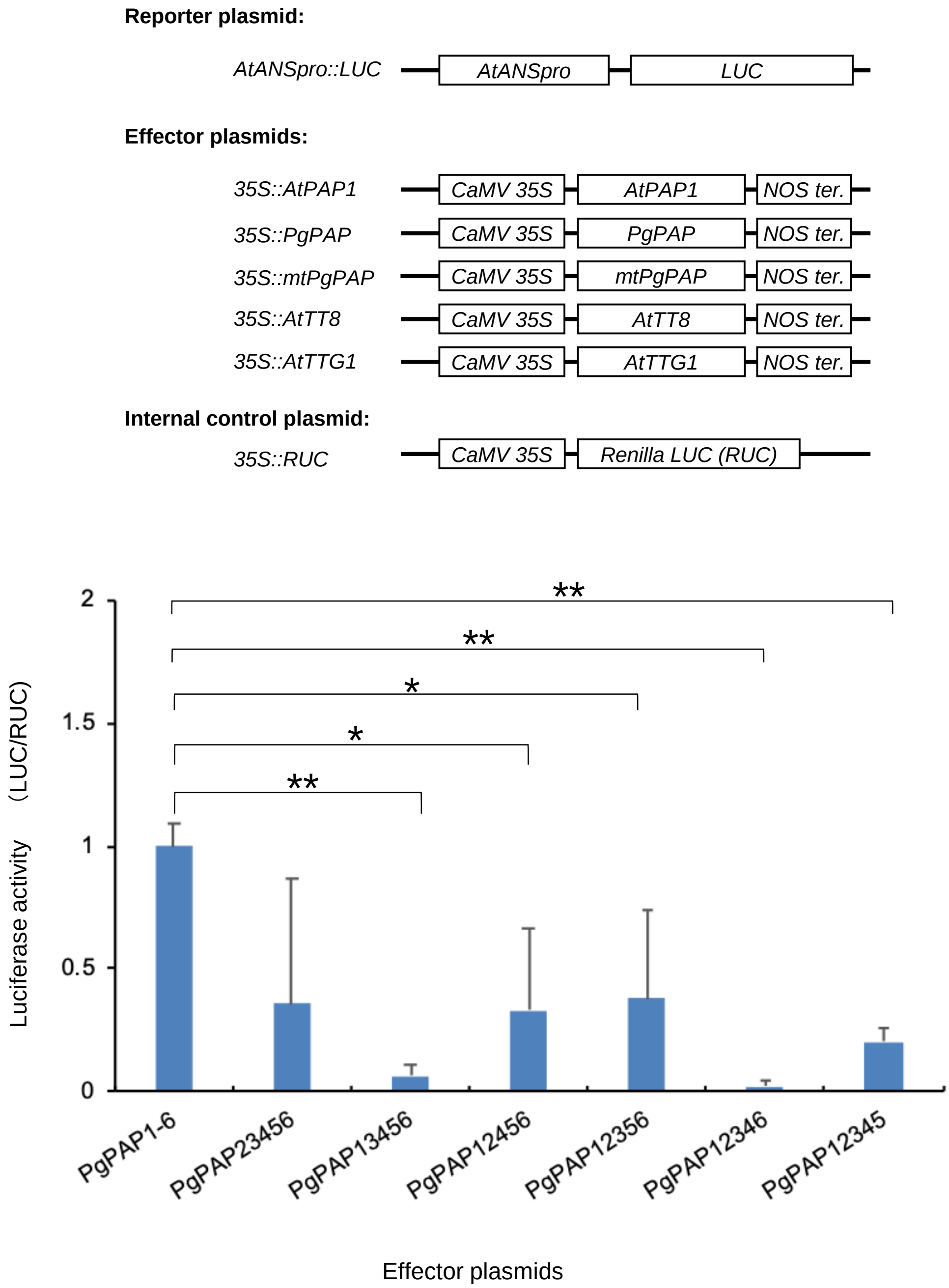

Fig. S6 Transactivation of *AtANS promoter* by PgPAP1-6 (mtPgPAP) replaced five of six amino acids common among PAPs of anthocyanin-producing plants. The upper panel shows the reporter and effector plasmid constructs (mean ± s.d., ***P* < 0.01, **P* < 0.05 by Student's t-test, n = 3).
